# High‐Resolution Multiplexed Sequencing of Single‐Cell Full‐length Transcriptome Via Combinational Barcoded Tn5 Transposon Insertion

**DOI:** 10.1002/advs.202516013

**Published:** 2025-11-11

**Authors:** Liyong He, Kaitong Dang, Qian Sun, Wenjia Wang, Wenbo Li, Wenyi Zhang, Kaiqiang Ye, Handong Wang, Zhengyue Li, Yan Guo, Zheng Li, Chencheng Yao, Peng Li, Yan Huang, Xiangwei Zhao

**Affiliations:** ^1^ State Key Laboratory of Digital Medical Engineering School of Biological Science & Medical Engineering Southeast University Nanjing 211189 China; ^2^ State Key Laboratory of Reproductive Medicine and Offspring Health Nanjing Medical University Nanjing 211166 China; ^3^ Department of Andrology Center for Men's Health Department of ART Institute of Urology Urologic Medical Center Shanghai Key Laboratory of Reproductive Medicine Shanghai General Hospital Shanghai Jiao Tong University School of Medicine Shanghai 200080 China; ^4^ Feinberg School of Medicine Northwestern University Chicago IL 60611 USA; ^5^ School of Biomedical Engineering Shanghai Jiao Tong University Shanghai 200242 China; ^6^ The Affiliated Taizhou People's Hospital of Nanjing Medical University Taizhou School of Clinical Medicine Nanjing Medical University Taizhou 225300 China

**Keywords:** alternative splicing, barcoded Tn5 transposase, full‐length transcriptome, multiplexed, single cell, spermatogenesis

## Abstract

The technological advancements in single‐cell transcriptome analysis make significant progress in both depth and breadth. However, balancing the cell analysis throughput with full‐length transcript coverage remains a persistent challenge. Here, CBTi‐seq (Combinational Barcoded Tn5 Transposon Insertion sequencing) is reported, leveraging Tn5 transposase‐mediated molecular assembly of combinatorial barcodes and unique molecular identifiers (UMIs) to enable high‐resolution multiplexed sequencing of the full‐length transcriptome in single cells. This approach achieves molecular resolution by end‐to‐end sequencing, enabling unambiguous reconstruction of splice variants and structural variations with base‐pair precision. The design of orthogonal combination barcode Tn5 reduces DNA barcode diversity while enhancing multiplexing flexibility, and Tn5‐delivered UMIs insertion eliminates read bias, providing accurately quantifies transcript abundance through the tagging of each fragment. The method is compatible with both single‐cell and spatially resolved tissue microenvironment. Compared with commercial terminal library and other full‐length sequencing methods, CBTi‐seq achieves superior sensitivity and resolution while significantly reducing costs and work time (≈5 h). Moreover, cell‐type‐specific alternative splicing patterns are robustly identified in both gene‐edited cells and human testicular cells, leveraging this high‐resolution capability to further reveal modality dynamic events and isoform switching independent of gene expression changes during spermatogenesis with the potential to reproductive development and diagnostic treatment.

## Graphical abstract

CBTi‐seq is reported, leveraging Tn5 transposase‐mediated molecular assembly of combinatorial barcodes and UMIs for high‐resolution multiplexed sequencing of the full‐length transcriptome in single cells. This approach achieves molecular resolution by end‐to‐end sequencing, enabling reconstruction of splice variants and accurate quantification of transcript abundance. CBTi‐seq offers superior sensitivity and resolution, significantly reducing costs (≈0.7 USD cell^−1^) and fast work time (≈5 h).

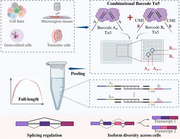

## 1 Introduction

Single‐cell RNA sequencing (scRNA‐seq) has provided a powerful tool in biology research to reveal the heterogeneity of complex samples and discover novel cell types in the past decade.^[^ ^1^, ^2^, ^3^, ^4^, ^5^ ^]^ Continued advances in molecular barcoding techniques and integration of droplet microfluidic‐/microwell array‐based platforms allow gene expression profiling across thousands to millions of single cells.^[^ ^6^, ^7^, ^8^, ^9^, ^10^ ^]^ However, most high‐throughput scRNA‐seq methods rely on barcoded oligo‐dT primers to capture and initiate reverse transcription (RT) from the poly(A) tail of mRNAs and convert the barcode sequences to the RNA end. These methods introduce inherent technical limitations, only detect a short part of the RNA (≈400–600 base pairs) adjacent to the end of the transcripts, resulting in obvious terminal bias. Although such bias won't perturb the result of profiling differential gene expression in high numbers of cells, the limited coverage of transcripts makes it difficult to reveal structure‐related information, such as alternative splicing (AS) events and allelic variants.^[^ ^11^, ^12^, ^13^ ^]^

In contrast, high‐resolution full‐length scRNA‐seq achieves molecular resolution by end‐to‐end sequencing, enabling unambiguous reconstruction of splice variants, allele‐specific expression, and structural variations with base‐pair precision.^[^ ^14^ ^]^ Smart‐seq2^[^ ^15^ ^]^ has been considered a “scRNA‐seq ruler” method to achieve full‐length transcriptome coverage for many years with high sensitivity, robustness and capacity of AS analyzing, but limited by quantification accuracy due to the lack of a unique molecular identifier (UMI). Although the optimized methods such as the Smart‐seq3^[^ ^16^ ^]^ protocol, has been the incorporation of UMI, there are still challenges of low cellular throughput, time‐consuming and cost inefficiency. Overall, a persistent challenge with current scRNA‐seq techniques is the balance between the improvement of throughput and the achievement of full‐length transcript coverage.^[^ ^17^ ^]^ Until recently, a novel technology called “VASA‐seq” that enables high‐throughput total RNA sequencing in single cells and provides full‐length coverage under the short‐read sequencing platform.^[^ ^18^ ^]^ Nonetheless, the method requires repetitive droplet generation and delicate pico‐injection operation, making it complicated for most general laboratories and preventing the broad application. To overcome these challenges, we sought to develop a high‐sensitive, low‐cost, and versatile method for multiplex, scalable transcriptome full‐length sequencing in single cells.

In this study, we report CBTi‐seq, a high‐resolution multiplexed sequencing of single‐cell full‐length transcriptome library construction protocol based on the insertion of Barcode Tn5 transposon with high sensitivity and scalability. Notably, the method reduces the demand of DNA barcodes diversity and increases the flexibility of multiplexing through the orthogonal combination barcoding of barcode Tn5 A_m_ and B_n_. The Tn5‐delivered unique molecular identifier (UMI) insertion eliminates read bias, providing precise quantitative analysis of the full‐length transcriptome through the tagging of each fragment. Meanwhile, we combine one‐step of RT and cDNA preamplification PCR (RT‐PCR) strategy to generate high yield and quality of full‐length cDNA in a shorter time and lower cost. The feasibility of CBTi‐seq in achieving multiplexed transcriptome full‐length coverage and its ability to identify AS events were demonstrated in the analysis of hundreds of single cells and microregion tissue samples from humans and mice. Finally, we applied adult human testicular cells from obstructive azoospermia (OA) donors and revealed the differential splicing patterns of different cell types during spermatogenesis. Taken together, CBTi‐seq enables sensitive and easily scalable multiplexed sequencing of the full‐length transcriptome from single cells in any laboratory without experience in scRNA‐seq.

## 2 Results

### 2.1 Performance Validation of Combinational Barcode Tn5 transposome

**Figure 1:**
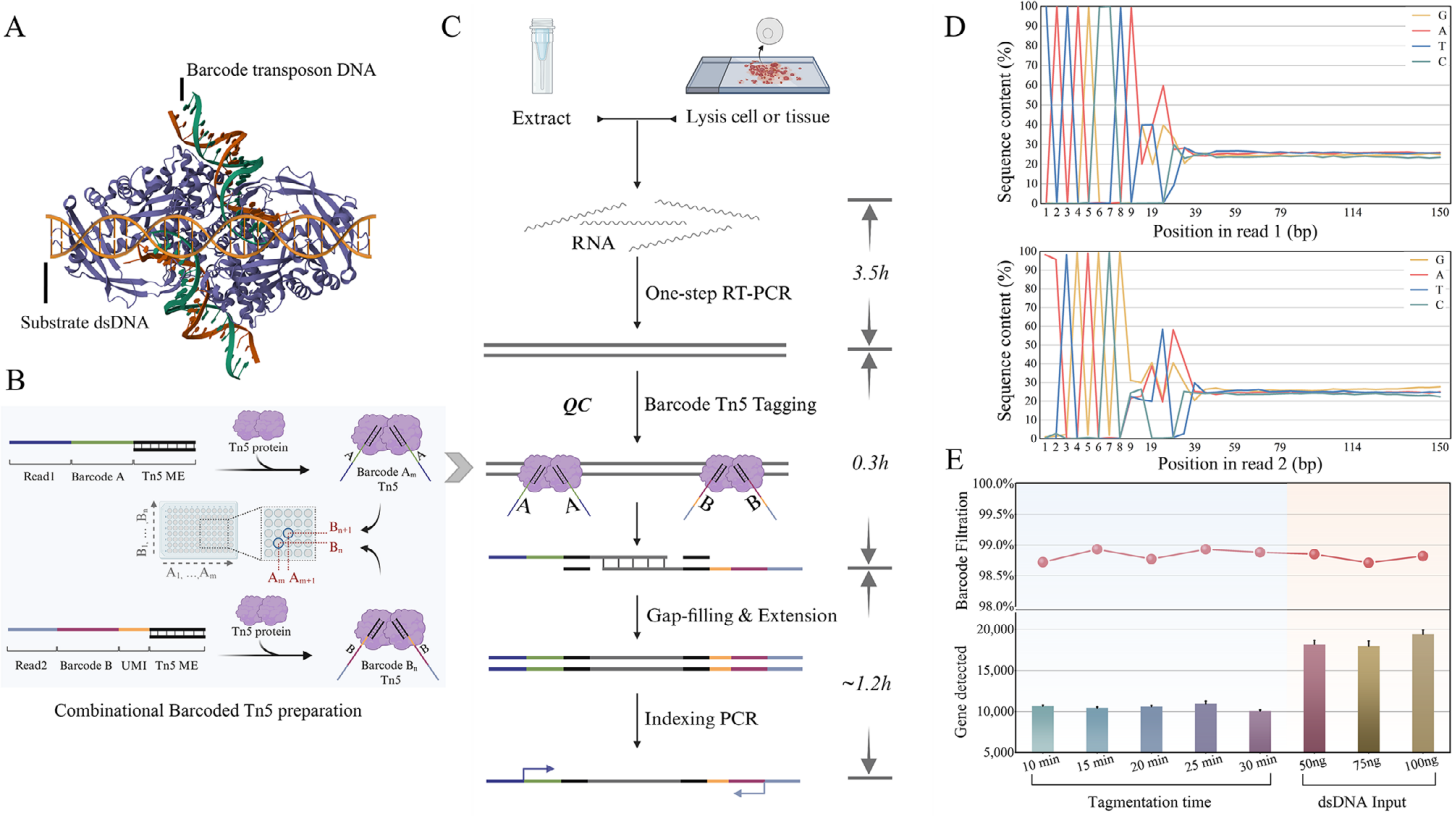
Overview of the tagmentation activity of Barcode Tn5 transposome on dsDNA and CBTi‐seq workflow. A) Model of docking of double‐stranded in predicted substrate binding sites of barcode Tn5. B) The principle of orthogonal combination Barcode Tn5 transposome preparation. C) Workflow of sequencing library preparation by CBTi‐seq. The input can be a lysed single cell and microregion tissue or extracted bulk RNA. After RT‐PCR with oligo‐dT primer, the preamplified full‐length dsDNA is directly tagmented by combinational Barcode Tn5 transposase, followed by gap‐filling & extension and indexing PCR. D) Per base sequence content according to read1 (top panel) and read2 (bottom panel) of FastQC. The abscissa represents the position of the base on each read, and the ordinate represents the percentage of the number of per base in the corresponding position. The first 8 bp specific types of bases was almost 100%, and the subsequent A/T and C/G reads were evenly distributed at 25%. E) Results of barcode filtrations and gene detected under different tagmentation time (10–30 min, 50 ng dsDNA input) and dsDNA input (50–100 ng, 20 min of tagmentation time). Top panel: Dot plot of barcode filtrations for all experiments (*n* = 3), each of the group shows>98.5% barcode readout; Bottom panel: Bars of gene detected for all experiments (*n* = 3), an average of ≈10 000–20 000 genes detection. All experiments used dsDNA are obtained from the RT‐PCR of H9 total RNA.

We first depicted a visual schematic model of barcode double‐stranded DNA (dsDNA) docking at the predicted substrate binding sites of Tn5 (**Figure** **1**A). The principle of Barcode Tn5 transposome preparation is shown in Figure 1B, Mosaic End sequence (ME) as the target site of Tn5 transposon, which is first hybridized to form ME‐A_m_ and ME‐B_n_ duplex adaptor, respectively. Then they are specifically inserted into Tn5 transposase to finally form Barcode A_m_ Tn5 and Barcode B_n_ Tn5 transposomes. Of note, the A_m_ sequence consists of Read 1, an 8‐nt barcode A_m_ index, and Tn5 ME. Similarly, the B_n_ sequence consists of Read 2, an 8‐nt barcode B_n_ index, a UMI, and Tn5 ME, where the UMI of each fragment facilitates more precise quantification of gene expression level. Unlike most of the commercial Tn5 transposase products, CBTi‐seq adopts the same adaptor in the Barcode Tn5 transposase, which allows for a more flexible combination without compromising reaction efficiency. Importantly, using this orthogonal combination barcoding, a multiplexed RNA‐seq library with m × n samples required only m + n types of Barcodes Tn5 transposome rather than m × n types required in traditional barcoding strategies, and both have the same tagmentation efficiency (Figure S1, Supporting Information).

To validate the feasibility and performance of Barcode Tn5 transposome, we designed two experiments for the gradient tagmentation time (10–30 min) and dsDNA input amount (50–100 ng). The dsDNA was obtained according to the Smart‐seq2 protocol using RNA extracted from hESC H9 cells, and the length was ≈2000 bp. Fragment analysis of the tagmented dsDNA ranging from ≈300–500 bp shows that the tagmentation time and input amount was positively correlated to the amount of fragment products but independent for the distribution (Figure S2, Supporting Information). Subsequently, the sequencing library of an individual sample was constructed according to the workflow of Figure 1C, the result of per base sequence content showed that the detection rate for the first 8 bp specific types of bases was almost 100% regardless of Read1 or Read2, demonstrating the Barcode Tn5 transposase successfully fragments the dsDNA and inserts the barcode sequence into the end of the product (Figure 1D). In addition, the barcode filtrations and gene detected results from all experiments showed >98.5% barcode readout and corresponded to ≈10 000–20 000 genes detected, directly proving that almost all sequencing reads contained correct barcode sequences and had considerable detection ability (Figure 1E).

Moreover, Tn5 transposase is known to not only mediate the transposition of dsDNA, but directly tagmentation of RNA/DNA heteroduplexes such as SHERRY^[^ ^19^ ^]^/SHERRY2.^[^ ^20^ ^]^ To explore whether our barcode Tn5 transposome could bind to RNA/DNA hybrids and construct downstream RNA‐seq library, we tested the performance with 10, 50 and 100 pg of H9 RNA inputs, each for three replicates (Figure S3, Supporting Information). Similarly, we observed more than 97% of barcode filtration in either experiment, indicating successful insertion into RNA/DNA hybrids and sequencing of barcode reads. Meanwhile, with the increase of RNA input, the uniquely mapping rate, transcript and gene count showed an increasing trend. Nevertheless, the low initial amount of RNA input analyzed a much lower number of genes than other scRNA‐seq methods. We reasoned that the structure of RNA/DNA hybrids were instability and extensive amplification can lead to bias, resulting in insufficient identification at low inputs.

As shown in above, we firmly chose to use pre‐amplified robust dsDNA to react with combination barcoding of Barcode Tn5 transposome under CBTi‐seq workflow, and demonstrated excellent tagmentation efficiency and decoding capability.

### 2.2 CBTi‐seq for full‐length sequencing library construction of independent sample

We tried to combine the reported one‐step RT‐PCR method^[^ ^21^ ^]^ with barcode Tn5 transposome technology to establish a simple, time‐saving and robust CBTi‐seq workflow. First, we evaluated and optimized the number of PCR cycles, reverse transcriptase, impact of crowding agents, and stop tagmentation buffer with 10 pg total RNA as input (Figure S4, Supporting Information). We optimized the number of PCR cycles and observed that a satisfactory cDNA yield and gene detected was obtained using 20‐cycle PCR and reaching plateau. For the RT step, the use of SuperScript IV and Maxima H reverse transcriptase allowed to detect more genes than the use of SMARTScribe. Meanwhile, SuperScript IV showed a slight improvement over Maxima H, but needs a higher reagent cost. The addition of PEG8000 seems not to be necessary during RT reaction. To explore the optimal stop Buffer of Tn5 transposase termination reaction, four different comparative groups (Table S1, Supporting Information) were performed for analysis. Within comprehensive evaluation of indicators including barcode filtration, uniquely mapping rate, sequencing saturation rate, gene body coverage heatmap, gene and transcript number, we finally chosen the most friendly and sensitive S‐2 strategy as the termination buffer (Figure S4C–F, Supporting Information).

**Figure 2:**
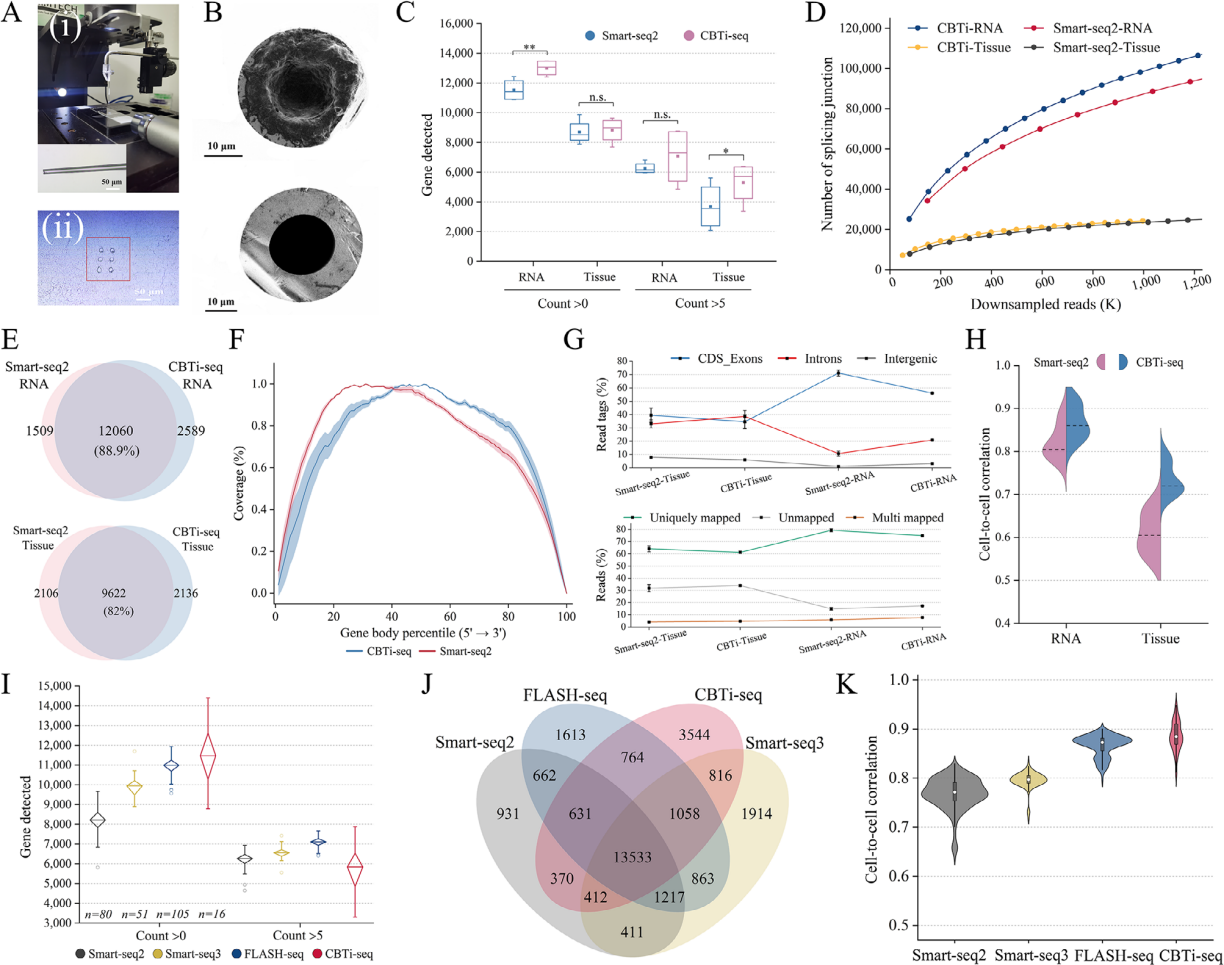
Comparison of performance between CBTi‐seq and other full‐length sequencing methods. A) microneedle sampling system for sampling micro‐region tissue samples. i) Schematic diagram of the sampling platform. The inset microscopic image is a glass capillary microneedle with a diameter of <30 µm. ii) Microscopic image of mouse brain tissue section after microneedle sampling. The sampled ROI points are in the red wire frame. Scale bar: 50 µm. B) Microneedles before (top panel) and after (bottom panel) tissue sample transfer is characterized by SEM images. Scale bar: 10 µm. C) Comparison of sensitivity to detect genes in extracted H9 RNA (*n* = 5) and tissue sample(*n* = 5) processed with CBTi‐seq and Smart‐seq2 method. Gene detection threshold was set to >0 or >5 reads. ^***^ *p* < 0.001, ^**^ *p* < 0.01, ^*^ *p* < 0.05. n.s.: no significant. D) The number of detected splicing junctions in RNA and tissue, for each method, is plotted against the number of reads per sample across different downsampling thresholds. E) A Venn diagram shows the overlap of detected genes by CBTi‐seq and Smart‐seq2 in the six replicates of RNA (top) or tissue sample (bottom). F) Gene body coverage averaged over the RNA sample sequenced with each method. Shown is the mean coverage of CBTi‐seq (blue) and Smart‐seq2 (red) shaded by the standard deviation. G) The top panel shows the percentage of read tags mapped to CDS exons, intronic or intergenic features in RNA and tissue. Bottom panel shows mapping statistics with the percentage of uniquely mapped, multimapped or unmapped reads for each method. H) Reproducibility analysis in gene expression quantification across RNA and tissue for each method. Shown are adjusted r values for all pairwise cell‐to‐cell correlations. The black dashed line represents the median value. I) Number of genes detected in HEK293T cells processed with Smart‐seq2 (*n* =  80), Smart‐seq3 (*n* =  51), FLASH‐seq (*n* =  105), and CBTi‐seq (*n* =  16) at two read thresholds, with reads downsampled to 500 000 (= 500K) raw reads. J) A Venn diagram shows the overlap of detected genes by CBTi‐seq and other methods in HEK293T cells (*n* = 16 for each method). K) Cell‐to‐cell Pearson correlations among gene expression in Smart‐seq2 (*n* = 80), Smart‐seq3 (*n* = 51), FLASH‐seq (*n* = 105), and CBTi‐seq (*n* =  16). The box plot shown in D,I) shows the median, first and third quartiles as a box, and the whiskers indicate the most extreme data point within 1.5 lengths of the box. The white dots in the violin K) plot represent the median values.

Under optimized conditions, we expected CBTi‐seq to exhibit similar robustness and flexibility comparable to that of Smart‐seq2 in full‐length transcriptome characterization. To illustrate this, we compared optimized CBTi‐seq with Smart‐seq2 using 10 pg extract RNA and single‐cell resolution mouse brain micro‐tissues, respectively. Remarkably, all micro‐region tissue samples were obtained using a home‐built microneedle sampling system^[^ ^22^ ^]^ (**Figure** **2**A(i)). The region of interest (ROI) on the tissue section was mechanically collected using a bore needle with a diameter of <30 µm, and then deposited into the PCR tube (Figure 2A(ii)). To verify that the samples were sufficiently transferred from the needle, we characterized the needles before and after sample transfer by scanning electron microscopy (SEM) images. As shown in Figure 2B, compared to before transfer, there was a significant decrease of tissue samples on the needle after deposition transfer, leaving only negligible trace residues, which strongly proved the successful collection of samples and reliability of this sampling platform.

After that, we employed the above two types of samples based on CBTi‐seq and Smart‐seq2 methods to evaluate the performance in transcriptome analysis (*n* = 5 for extract RNA and micro‐tissue, respectively). Profiling gene expression with CBTi‐seq significantly improved the gene detection ability compared to Smart‐seq2 under different read thresholds (Figure 2C), and regardless of the sequencing depth (Figure 2D). The higher sensitivity of the method facilitates the capture of a greater inform about isoforms and genes. The substantial overlap (from 82% to 88.9%) of co‐detected genes was observed in Venn diagrams (Figure 2E), showing the high consistency between methods and the reliability of CBTi‐seq. Besides, the CBTi‐seq method exhibited homogeneous full‐length gene‐body coverage and no obvious 3′‐ or 5′‐end bias observed, whereas Smart‐seq2 showed mild 5′‐end bias (Figure 2F). We further assessed the distribution of sequenced reads across known genome features (Figure 2G). Overall, the sequencing results revealed high quality libraries where the read distribution and uniquely mapped rate (>70% for RNA and >60% for tissue) of CBTi‐seq were comparable to those of smart‐seq2, regardless of sample types. Notably, we observed a higher proportion of intronic reads in CBTi‐seq than smart‐seq2 method, indicating more enriched nascent RNA was detected. In addition, the result in Figure 2H shows CBTi‐seq with improved correlation coefficients in gene expression profiles among different samples (Pearson's r = 0.85 vs 0.8 for RNA and 0.73 vs 0.61 for tissue), confirming the high quantitative reproducibility of this method. The main reason may be that CBTi‐seq simplifies the tedious RT‐PCR steps in traditional RNA‐seq library construction, and the insertion of UMI read contribute to improve the accuracy of error correction during sequencing.

Furthermore, we evaluated the overall performance of CBTi‐seq, Smart‐seq2/3 and FLASH‐seq using HEK293 cells (the data of smart‐seq2/3 and FLASH‐seq were obtained by the ref.)^[^ ^21^ ^]^ As shown in Figure 2I, CBTi‐seq detects a significant number of competitive genes in HEK293T cells under two read thresholds., with reads downsampled to 500K raw reads. Subsequently, we randomly selected 16 HEK293T single‐cell data from each method for the analysis of gene co‐detection. Figure 2J shows that a large number of gene types were jointly identified, which proves the accuracy of our method. Compared with Smart‐seq 2/3, CBTi‐seq significantly improved the cell‐to‐cell correlation in the gene expression profile (Figure 2K), and was comparable to the level of FLASH‐seq. We also compared the performance of these methods in detail through a list (Table S2, Supporting Information), including the workflow time, quantitative ability at the gene/transcript level, multiplexed library construction capabilities, and the experimental costs under 96‐ or 384‐ samples. We conclude that CBTi‐seq has increased sensitivity, accuracy and robustness compared to Smart‐seq2/3, but with a faster (within 5 h), easy to operate and lower cost. Meanwhile, compared with FLASH‐seq, it demonstrates competitive analytical performance and workflow time, but also possesses the capabilities of multiplexed library construction and subtype structure quantification at the transcript level that FLASH‐seq lacks.

### 2.3 CBTi‐seq for Multiplexed Transcriptome Full‐Length Sequencing Library Construction

**Figure 3:**
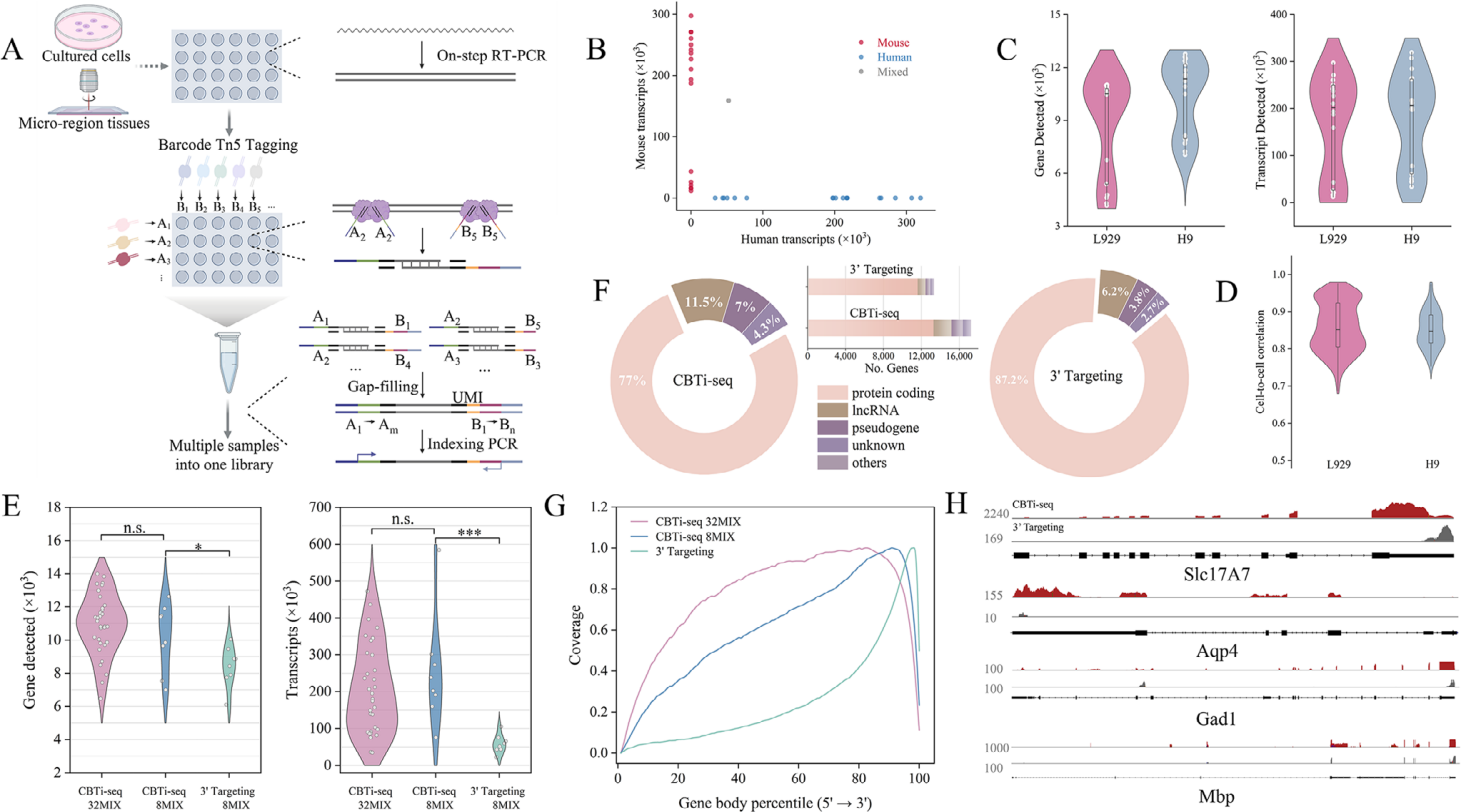
The workflow and performance of CBTi‐seq for multiplexed transcriptome full‐length sequencing. A) Workflow of multiplexed transcriptome full‐length sequencing library construction of CBTi‐seq. Input is compatible with single cells and micro‐regional tissues. After the cDNA (one‐step RT‐PCR for synthesis) of each independent sample was tagmented by a unique combinatorial Barcode “A_m_+B_n_” Tn5 transposome, all products were collected into one reaction tube by purification for subsequent extension and multiplexed library building. B) Mixed‐species cross‐contamination experiment for CBTi‐seq was carried out using H9 cells (human) and L929 fibroblast cells (mouse). Barcodes with more than 20% of detected belonging to the other species were considered doublets/ mixed (gray). The remainder were assigned to either mouse (red) or human (blue). C). Sensitivity of genes detected (left panel) and transcripts (right panel) in L929 cells (*n* = 18) and H9 cells (*n* = 18) from the mixed‐species experiment. D) Cell‐to‐cell correlation showing the quantitative reproducibility of CBTi‐seq between single cells. E) Sequencing performance comparison with CBTi‐seq with 3′ Targeting kit protocol at saturating read depths using microregion tissue (diameter of ≈20 µm). An average of 10931 genes and 207163 transcripts are detected in CBTi‐seq 32MIX (*n* = 32), average of 10251 genes and 253119 transcripts in CBTi‐seq 8MIX (*n* = 8), comparable to 8426 genes and 58105 transcripts in 3′ Targeting kit (*n* = 8). ^***^ *p* < 0.001, ^**^ *p* < 0.01, ^*^ *p* < 0.05. n.s.: no significant. F) Distribution of mapped reads across RNA types in mouse brain tissues using two methods. Percentage of total reads mapped to each RNA type. Bar plots showing the number of genes detected across RNA types. G) Gene body coverage with CBTi‐seq (32MIX and 8MIX) and 3′ Targeting method (8MIX) showing the multiplexed CBTi‐seq could compatible with the full‐length sequencing coverage. H) IGV tracks showing the coverage of four representative transcripts processed with CBTi‐seq and 3′ Targeting kit. Red: data of CBTi‐seq; Gray: data of 3′ Targeting; Black lines show the structure of selected genes that include or exclude the exon.

Considering that CBTi‐seq carries combinatorial barcoding, the RNA‐seq library preparation after Tn5 tagmentation can not only achieve typical full‐length sequencing, but also allow high‐throughput single‐cell transcriptome profiling by pooling multiple tagmentation products of single cells into one library preparation, enabling both scalability and cost‐effectiveness. The workflow of multiplexed transcriptome full‐length sequencing library construction of CBTi‐seq is described in **Figure** **3**A. First, a panel of single cells or micro‐region tissues is deposited into different tubes/microwells containing lysis buffer. Following sample lysed, mRNAs are captured by CBTi‐oligo (dT) primers and for one‐step RT‐PCR reaction. Next, each full cDNAs amplified are diluted to the same concentration and combinatorial Barcode “A_m_+B_n_” Tn5 transposome‐based tagging. Finally, the DNA fragments of all samples, each inserted with a unique combination barcoding, are pooled and purified into a single reaction tube for gap‐filling & extension and sequencing library.

To simplify the pooling protocol, we explored the use of 1.5 × ratio Ampure XP beads to purify and collect the mixed tagmented products while also terminating the tagmentation of Tn5 transposase without adding extra stop buffer. We designed a comparative experiment between direct pooling (DP, *n* = 6) and purification pooling (PP, *n* = 9) of mixed samples (Figure S5, Supporting Information). In theory, the barcode filtration corresponding to DP and PP should be 16.7% (1/6) and 11.1% (1/9), and we found after sequencing, the average 14.09 ± 10.3% and 10.37 ± 3.9% barcode filtration of DP and PP, respectively, indicating that the bias introduced in PCR library construction by DP was relatively large, with not conducive to downstream data analysis (Figure S5A, Supporting Information). Besides, the gene and transcript expression level, and quantified correlation were further used to assess the performance (Figure S5B–E, Supporting Information). These results demonstrate that the PP method (1.5 × Ampure XP beads to purify and collect) allows to produce less bias and detects more gene expression information.

To verify that the CBTi‐seq workflow remained the accuracy of each gene origin, we profiled a species‐mixing experiment with mouse L929 fibroblast cells and human hESC H9 cells, which showed the nearly all of the combinatorial barcodes uniquely aligned to either the human or mouse transcriptome, indicating highly species‐specific and minimal cross‐contamination between the multiplexed sequencing library construction (Figure 3B). Then, the L929 and H9 cell datasets were analyzed to determine the gene detection sensitivity of this workflow in single cells. CBTi‐seq exhibited the high sensitivity, with a broad spectrum of 10 021 (L929 cells) and 11461 (H9 cells) detected genes per cell, corresponding to an average of ≈172 557 transcripts in L929 cells and ≈185 482 transcripts in H9 cells (Figure 3C). Cell‐to‐cell correlation indicated the good quantitative reproducibility of CBTi‐seq between single cells (r = 0.865 in L929 cells and r = 0.852 in H9 cells) (Figure 3D; Figure S6, Supporting Information).

Additionally, to demonstrate the scalability and future potential of CBTi‐seq for ROI unbiased spatially transcriptome, we sampled two sets of micro‐region tissue samples with near single‐cell resolution (≈20 µm in diameter) on thin mouse brain sections for multiplexed full‐length sequencing, called 8‐MIX and 32‐MIX tissue samples, respectively, and further compared CBTi‐seq to the widely used 3′ specific amplification library building commercial kit (TruePrep®DNA Library Prep Kit, 3′ targeting). We detected an average of ∼207 163 transcripts from ∼10 931 genes in 32‐MIX, and ∼253 119 transcripts from ∼10 251 genes in 8‐MIX at saturating read depths (Figure 3E). Importantly, there were no significant decrease in the detection level due to the increase of samples, indicating that CBTi‐seq has good compatibility for higher throughput. The 3′ targeting‐based workflow of 8MIX detected ∼58 105 transcripts from ∼8426 genes, showing lower sensitivity than CBTi‐seq. Based on the proportion of mapped reads across RNA types, protein‐coding genes showed the most highly detected biotype among these methods (Figure 3F). Interestingly, we observed CBTi‐seq workflow detected more than twice as many long non‐coding RNAs (lncRNAs) as 3′ targeting, and lncRNAs have been proven to play vital roles in the regulation of translation, metabolism and signaling.^[^ ^23^ ^]^

Moreover, the 3′ targeting technique had a large bias toward the 3′ end. In contrast, the CBTi‐seq multiplexed workflow exhibited a higher coverage in the bias of 5′ end except for 3′ end reads, which facilitated the unbiased capture of splicing junctions. However, we also found that CBTi‐seq‐8MIX shows a certain 3′ bias, which may be due to the RNA degradation caused by the long‐term storage of tissue samples. To prove this point, we sequenced and analyzed the samples under different storage times respectively. The results confirmed that the coverage bias continuously increased with the increase of sample storage time (Figure S7, Supporting Information). We next used an integrated genome viewer (IGV) visualization of selected genes (Slc17A7, Gad1, Aqp4, and Mbp) from tissues processed with CBTi‐seq and 3′ targeting, respectively (Figure 3H; Figure S8, Supporting Information). All visualization results indicated that the full‐length sequencing of CBTi‐seq has the potential to accurately identify alternative splicing.

### 2.4 CBTi‐seq Profiling Spatially Resolved Single‐Cell Transcriptomics in Mouse Brain Functional Areas

**Figure 4:**
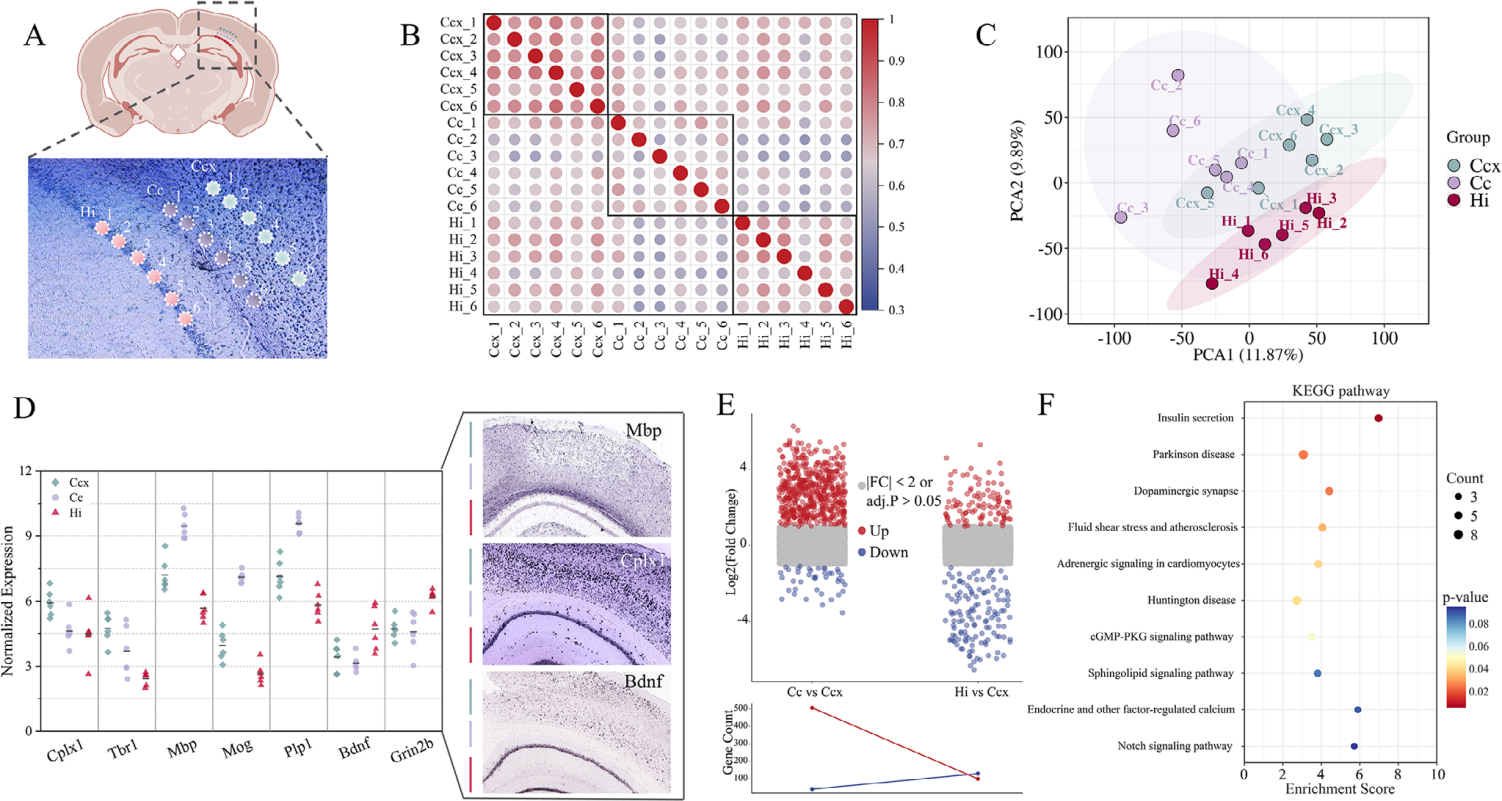
CBTi‐seq reveals the spatial heterogeneity of mouse brain functional regions at the single‐cell resolution. A) Experimental design. Single‐cell‐resolution micro‐region sampling was performed on three functional regions within the H&E‐stained mouse brain section. Sampled tissues from each functional region (*n* = 6 biological replicates) were processed through CBTi‐seq for multiplexed library construction and sequencing. Ccx: cerebral cortex; Cc: corpus callosum; Hi: hippocampus. B) Heatmap showing Pearson's correlation coefficient of the normalized gene expressions between 18 samples from Ccx, Cc, and Hi functional regions. The color bar represents Pearson's correlation coefficient. C) PCA analysis of the three groups of Ccx, Cc, and Hi samples. D) A panel of selected differentially expressed genes among the three regions (left) and the Allen Brain Atlas’ ISH results were verified as references (right). E) Multi‐group difference analysis volcano plot showing up‐ and down‐regulated genes (blue: down‐regulated, red: up‐regulated) across Cc and Hi compared to Ccx, with the line chart showing the number of differentially expressed gene counts. F) The enriched signaling pathways of Hi compared to Ccx via KEGG pathway analysis.

To evaluate the potential utility of the CBTi‐seq for spatially resolved transcriptomics tissue analysis, we performed a proof‐of‐concept experiment by microneedle sampling and analyzing three typical mouse brain regions (cerebral cortex (Ccx), corpus callosum (Cc), and hippocampus (Hi)) under the specific zone. Based on the HE‐stained region image depicted in **Figure** **4**A, tissue samples were collected as circular regions with a diameter of ≈15 µm, corresponding to an area of ≈177 µm^2^. The spatial distances (center to center) were from 40 to 200 µm within the same brain region, and from 150 to 350 µm across different brain regions. The pairwise correlation analysis of quantitative gene expression quantification data from 18 samples showed a relatively high correlation coefficient between biological replicates of the same brain micro‐regions, whereas Cc had lower correlations with the other two regions. This difference is also consistent with the morphology of the brain tissue (Figure 4B). After data normalization, we used principal component analysis (PCA) and hierarchical cluster analysis (HCA) to segregate the three brain tissue regions (Figure 4C; Figure S9, Supporting Information). Each of six replicates from the same region had a distinct cluster based on gene expression abundance.

The left of Figure 4D shows a panel of selected differentially expressed genes in each tissue region. Cplx1 and Tbr1 (a marker gene for the cortex) were enriched in Ccx region. The myelination of Cc axons is highly active during the development of the mouse brain. As expected, we found myelin‐related genes (Mbp, Mog, and Plp1) were highly expressed in the Cc region. In the Hi region, we observed the high expression of brain‐derived neurotrophic factor (Bdnf) and Grin2b. Bdnf plays an important role in several aspects of hippocampal neuronal plasticity and function.^[^ ^24^ ^]^ Similar to Bdnf, a previous study has demonstrated that the Grin2b gene is critical for neuronal migration, synaptogenesis, and cortical generation during brain development.^[^ ^25^ ^]^ Combined with the visualization images from the Allen Brain Atlas ISH, the specific mapping and differential expression of the selected gene (Mbp, Cplx1, and Bdnf) in different functional regions are further confirmed, which is consistent with our sequencing results (right of Figure 4D).

Furthermore, we employed a volcano plot to evaluate gene variance (padj <0.05, |log_2_FoldChage| > 1) across Cc and Hi compared to Ccx (Figure 4E). We identified 92 genes as significantly upregulated in the Hi region compared to the Ccx region. To evaluate the biological significance of these genes, we performed Kyoto Encyclopedia of Genes and Genomes (KEGG) to treat with gene enrichment, and the top ten enriched categories were listed in Figure 4F. The Hi‐enriched genes were mainly associated with Parkinson disease, Huntington disease, Insulin secretion, and Dopaminergic synapse. Overall, CBTi‐seq enables spatially resolved transcriptomics analysis in the tissue microenvironment, and holds great potential for revealing the spatial and temporal characteristics and functional heterogeneity of cells.

### 2.5 CBTi‐seq Validates Specialized Alternative Splicing Patterns in Gene‐Edited Cells

**Figure 5:**
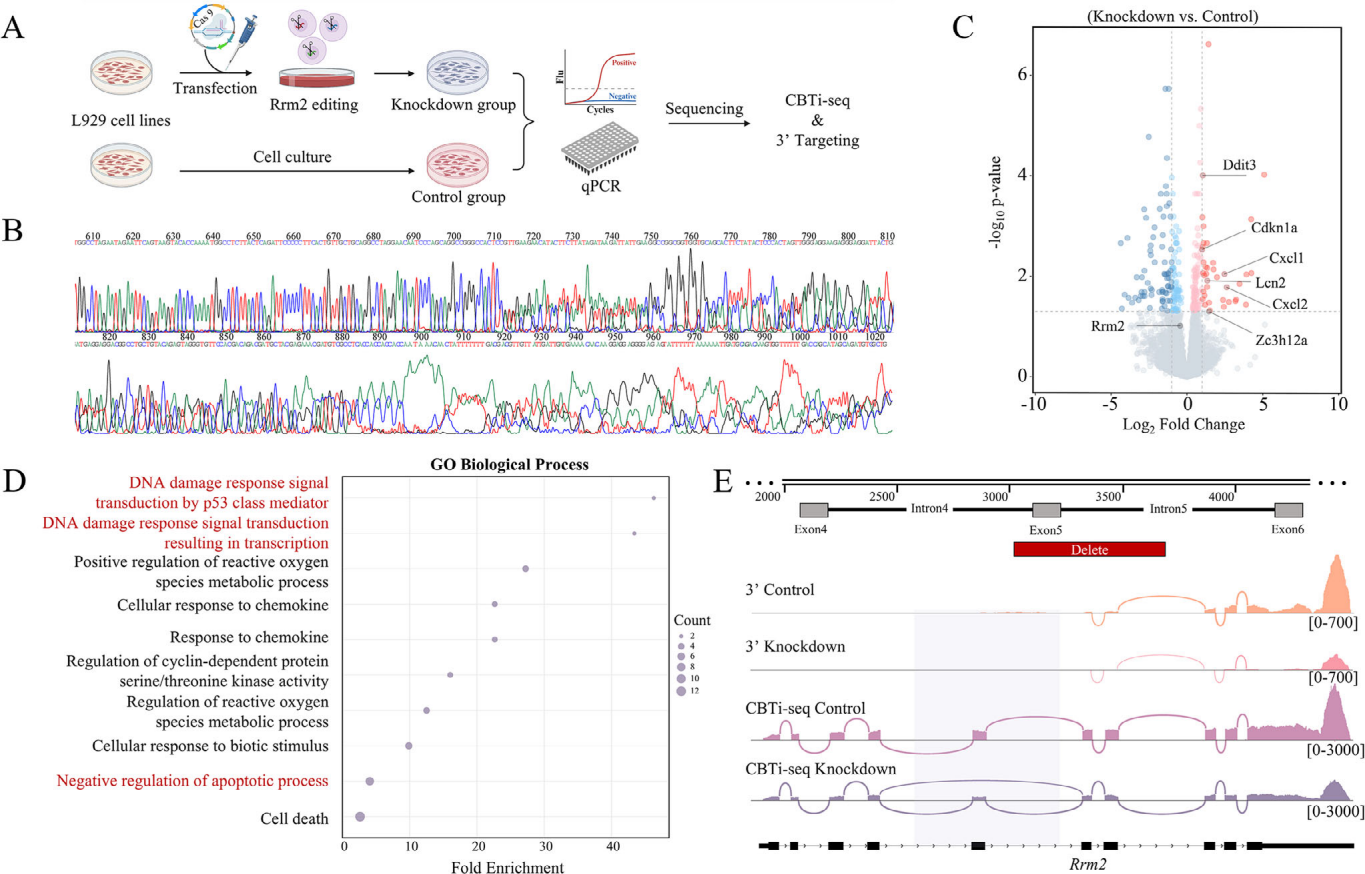
CBTi‐seq reveals the specialized alternative splicing patterns in gene‐edited cells. A) Experimental design. The constructed plasmid containing CRISPR‐Cas9 was transfected into L929 cells to establish Rrm2 gene‐knockdown and control cell groups. Then the two groups were validated and sequenced by CBTi‐seq and 3′ Targeting methods, respectively. B) The heterozygous peak plot from the sequencing result of the successful Rrm2‐knockdown cells were shown. C) Volcano plot showing 38 up‐regulated genes and 74 down‐regulated genes screened out in knockdown cell groups compared to control groups (adjusted p.adj <0.05, fold change >2). D) GO enrichment analysis of upregulated genes in knockdown cells. Biological process of “DNA damage” and “negative regulation of apoptotic process” are marked in red. E) CBTi‐seq revealed exon‐junction and alternative splicing patterns compared to 3′ Targeting in two groups. Up: The schematic diagram of genome editing (knocking out the Exon 5 region of Rrm2) with CRISPR‐Cas9. Down: Sashimi plot of sequencing data aligned at the Rrm2 locus. The lines represented the exon‐junction. Rrm2 exon 4 to exon 6 showed exon skipping in the knockdown groups by the analysis of CBTi‐seq. The range for normalized read counts is indicated between brackets.

To validate the capability of CBTi‐seq in detecting alternative splicing (AS) events, we performed genetic editing on L929 cell lines. As shown in **Figure** **5**A, the constructed plasmid vectors (Figure S10A, Supporting Information) were transfected into L929 cells to establish Rrm2‐knockdown and control cell groups. Heterozygous peak patterns in sequencing chromatograms confirmed successful Rrm2 knockout near the exon 5 region (Figure 5B). Principal component analysis (PCA) of single‐cell transcriptomic data demonstrated distinct clustering between knockdown and control groups (Figure S10C, Supporting Information). We next compared whether the same cell type captured obvious known functional differences before and after genetic editing. Comparative analysis revealed 38 significantly upregulated genes and 74 downregulated genes in the knockdown groups (Figure 5C; adjusted p.adj <0.05, |fold change| >2). Gene ontology (GO) enrichment analysis of upregulated genes, including Ddit3, Cdkn1a, Cxcl1, Lcn2, Cxcl2, and Zc3h12a, demonstrating that the knockdown group mainly experienced biological processes related to DNA damage response and negative regulation of apoptotic process (Figure 5D).

Moreover, we compared the ability to reveal cell‐specific splicing patterns between knockdown and control groups using CBTi‐seq and 3′ targeting methods. The number of detected genes showed no significant difference between the two methods, and the average number of genes exceeded 12 000 (Figure S10B, Supporting Information). However, gene body coverage distribution indicated differential genomic coverage patterns, which CBTi‐seq shows a uniform full‐length coverage (Figure S10D, Supporting Information). Sashimi plots further demonstrated that CBTi‐seq enabled comprehensive profiling of Rrm2 coding exons, whereas 3′ targeting was restricted to 3′‐end regions, resulting in substantial loss of exon junction information (Figure 5E). Notably, with base‐pair precision enabled by end‐to‐end sequencing, CBTi‐seq robustly identified pronounced exon skipping events spanning exon 4 to exon 6 in the knockdown group, demonstrating unambiguous reconstruction of splice variants at molecular resolution. Taken together, these results successfully validated partial Rrm2 knockout near exon 5 and underscored the superior capability of CBTi‐seq in detecting AS events and isoform‐specific structures.

### 2.6 CBTi‐seq Profiles Cell Type and Splicing Patterns in Adult Human Testicular Cells

**Figure 6:**
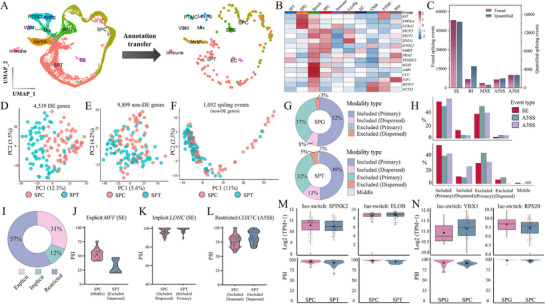
CBTi‐seq profiles cell type and splicing patterns in adult human testicular cells. A) Left panel: UMAP plots of the human testicular reference map. Cell types are indicated by color and inset text labels. UMAP, uniform manifold approximation and projection. Right panel: UMAP of 192 dissociated testicular cells from two human donors based on annotation transfer from reference map to CBTi‐seq data (*n* = 192). B) Heatmap of selected gene markers identified from CBTi‐seq, compared across all annotated cell types. The color key from blue to red indicates low to high gene expression levels, respectively. C) Mavel detected and quantified splicing events in testicular cells. D–F) PCA plots using D) differentially expressed genes, E) non‐differentially expressed genes, and F) all alternative splicing events from non‐differentially expressed genes. non‐DE, non‐differentially expressed. G) The proportion of each modality class in SPC and SPT cells. H) The proportion of each modality class by splicing event type in SPC and SPT cells. I) The proportion of each modality dynamic class, i.e., the type of changes in modality of alternative splicing events from SPC to SPT cells. J–L) Representative alternative splicing events of each modality, dynamic class J) explicit, K) implicit, and L) restricted. M,N) A representative gene and corresponding alternative splicing event for each gene‐splicing relationship class, M) isoform switching in SPC‐SPT cells, and N) isoform switching in SPG‐SPC cells.

Spermatogenesis is a highly complex and precisely regulated process. Moreover, alternative splicing (AS) is widely prevalent in the testis, but the regulations of AS in spermatogenesis is only little explored.^[^ ^26^ ^]^ As methodological validation, we performed random cell picking from two male donors with obstructive azoospermia (OA) and collected 192 adult testicular cells, with an average of 7583 expressed genes detected per cell (Figure S11, Supporting Information). By integrating CBTi‐seq data into the reference‐based published scRNA‐seq mapping,^[^ ^27^ ^]^ we could robustly assign nine cell types (**Figure** **6**A). These clusters could be further divided into two parts, germ cells and microenvironment somatic cells. The former included spermatogonia (SPG), spermatocyte (SPC) and spermatid (SPT); and the latter included endotheliocytes (EC), immune cells, vascular smooth muscle cells (VSM), peritubular myoid cells (PTMC), Sertoli cells, and Leydig cells. Heatmap in Figure 6B shows an average expression of cell‐type specific genes. This includes several of the well‐established markers for germ cells, such as *KIT*, *PIWIL4*, and *GFRA1* for SPG cells, *SYCP3*, *SYCP1*, *SPO11*, *OVOL2*, and *NME8* for SPC cells, and *PRM1*, *TXNDC2* for SPT cells.^[^ ^28^ ^]^ In addition, we found that *SOX9*, *AMH*, and *CLU* was significantly highly expressed in Sertoli cells, which was in close agreement with previous reports.^[^ ^28^ ^]^

To analyze the splicing patterns in adult human testicular cells, we performed AS analysis platform, Marvel,^[^ ^29^ ^]^ to characterize the splicing landscape and reveal biological insights during the spermatogenesis process. Figure 6C shows that Mavel detected and quantified over 80 000 and 30 000 splicing events in testicular cells, including skipped‐exons (SE), retained‐introns (RI), mutually exclusive exons (MXE), alternative 5 and 3 splice sites (A5SS and A3SS) splicing events. Next, we focused on the cellular gene expression and splicing dynamics during the spermatogenesis process. Among them, SPC and SPT cells are the functional transition periods of spermatogenesis and have significant clinical research significance. Differentially expressed genes could distinguish the cell types (Figure 6D), whereas non‐differentially expressed (non‐DE) genes could not separate the cell types (Figure 6E). Interestingly, differential splicing events from non‐DE genes could enhance the ability to delineate the two cell types (Figure 6F, 11% of PC1). We then explored percent spliced‐in (PSI) distributions (modalities) of individual AS events identified in SPC and SPT cells. Modality assignment can inform whether an individual isoform (included and excluded) or both isoforms (bimodal, middle, and multimodal) are expressed in a cell population. In addition, the Marvel platform could stratify the included and excluded modalities into primary and dispersed. The results indicated that each type of cell‐specific splicing event is assigned to different patterns (Figure 6G). Both cell types showed a high proportion of included and excluded modalities, while other modalities showed only ≈2%. Furthermore, the different proportions of selective splicing events in the modality types were also revealed (Figure 6H).

Next, we categorized differential AS events based on modality changes (explicit, implicit, and restricted) between SPC and SPT cells. Interestingly, we observed that the explicit modality changes only detected ≈31% of the splicing event differences (Figure 6I). This typical research modality often misses the majority of the differential selective splicing events. We have listed several genes that undergo explicit, implicit, and restricted modality changes in SPC and SPT cells, corresponding to *MFF*, *LDHC* and *COX7C* respectively (Figure 6J–L). MFF, as a mitochondrial fission factor, plays a crucial role in regulating the organization of mitochondria and the energy supply for sperm development during the complex developmental process,^[^ ^30^ ^]^ whereas LDHC and COX7C are closely related to the specific energy metabolism requirements during the reproductive process.^[^ ^31^, ^32^ ^]^ This is consistent with the reports in the literature that mitochondrial dysfunction is a major component of sperm dysfunction and male infertility.^[^ ^33^ ^]^ To explore the relationship between differential genes and changes in alternative splicing, we depicted several genes with isoform switching in SPC‐SPT cells (Figure 6M). This is defined as genes that showed differential splicing but non‐DE. For example, SPINK2, involved in the acrosomal biogenesis and sperm differentiation,^[^ ^34^ ^]^ showed no significant difference in gene expression but the mean PSI values deviated during the process from SPC to SPT. ELOB is localized in the nuclei of postmeiotic male germ cells and regulated post‐transcriptionally in the testis.^[^ ^35^ ^]^ It showed no significant gene expressed difference, and the average of PSI values in both cells were similar, but the overall PSI distribution for an SE event was changed from included primary in SPC to included dispersed in SPT cells (implicit modality change). Moreover, we also investigated isoform switching in SPG‐SPC cells (Figure 6N) and found that YBX1^[^ ^36^ ^]^ (involved in transcriptional regulation, mRNA stabilization, and translational repression) and RPS20^[^ ^37^ ^]^ (a housekeeping gene) conform to this regulatory relationship. Subsequently, we conducted a gene ontology (GO) analysis on the differentially spliced genes in the SPC‐SPG group and identified several significantly enriched pathways, including pathways related to RNA stabilization, gene translation and regulation, and organelle fission (Figure S12, Supporting Information). The coordinated action of these pathways may facilitate the smooth progression of SPC into meiosis. Overall, these results revealed that most PSI changes cannot be directly inferred from changes in gene expression, highlighting the value of differential splicing analysis in uncovering other differentially regulated genes.

## 3 Discussion

Single‐cell RNA sequencing technologies have provided new insights into revealing cellular complexity at unprecedented resolution. Meanwhile, the analysis of full‐length transcripts from single cells still remains limited in cellular throughput. Building on the powerful functionality and design flexibility of Tn5 transposase, we proposed a new method, CBTi‐seq, a tagmented insertion technology based on orthogonal combinatorial Barcode Tn5 transposase that enables multiplexed transcriptome full‐length sequencing. The high‐resolution nature of CBTi‐seq, achieved through end‐to‐end full‐length sequencing, provides unprecedented molecular resolution for single‐cell transcriptomics. This capability enables unambiguous reconstruction of splice variants and structural variations with base‐pair precision, which is pivotal for dissecting complex biological processes.

CBTi‐seq is a simple, sensitive, and cost‐effective method for gene expression profiling that compares favorably with previous methods. Comparison of CBTi‐seq with the commonly used Smart‐seq2 or Smart‐seq3 protocol for input at the single‐cell scale, showed comparable full‐length coverage, but more sensitive (detecting >20% more genes than Smart‐seq2 and >10% more than Smart‐seq3), less workflow time (≈9–10 h by Smart‐seq2/3), and higher quantitative performance. Besides, CBTi‐seq is fully suitable for multiplexed sequencing across varying sample numbers without significantly reducing the detection sensitivity. In addition, our method possesses the capabilities of multiplexed library construction and subtype structure quantification at the transcript level that Smart‐seq3 and FLASH‐seq lacks. Compared with the commercial 3′ Targeting TruePrep kit, CBTi‐seq showed more sensitive gene detection results in the sequencing of multiplexed samples, including protein‐coding and lncRNA genes. More importantly, with the advantage of full gene‐body coverage, CBTi‐seq facilitated the information about AS events in transcript‐level analysis, and we have visualized several marker genes from mouse brain tissue samples with different structural detection differences between the methods. In addition, we have identified cell‐type‐specific alternative splicing patterns in both gene‐edited cells and human testicular cells, further revealed modality dynamic events and isoform switching independent of gene expression changes during spermatogenesis. Furthermore, the whole CBTi‐seq workflow to sequencing library consists of only takes ≈5 h which is much less labor‐intensive and time‐saving than 3′ Targeting TruePrep kit (≈7 h). Moreover, compared with the other methods, the CBTi‐seq reagent cost is significantly lower while processing the same number of samples (Figure S13, Supporting Information). For example, in the multiplexed sequencing of 192 single cells from testicular tissue, the library construction cost of this method only needs to be ≈$144 USD, while the cost of the gold standard Smart‐Seq2 is as high as $8083 USD (≈53 times) and ≈$200 USD using Smart‐se3/FLASH‐seq.

Combining high‐resolution CBTi‐seq with a home‐built microneedle sampling platform holds the promise for dual‐scale mapping, including spatial precision at the micron level and molecular precision at the base‐pair level. In most scRNA‐seq studies, the tissue is homogenized and thousands of cells are used to create individual sequencing libraries, but lost the spatial location information of the cells in the context of the tissue. In contrast, microneedle & CBTi‐seq can identify cells of interest or micro‐regions of interest through morphology or staining markers, and the gene expression profiles can be traced back to their tissue locations, allowing for the mapping of local high‐resolution whole transcriptome spatial mapping. Compared to most LCM‐based methods, this sampling platform offers cost‐effectiveness, simplicity, high‐quality sample collection, and high resolution, Moreover, when collecting single‐cell resolution tissue samples, the success rate of this can reach up to 95%, which is much higher than that of the LCM platform under the same conditions, although it is currently limited by the inability to sample any shape.

Objectively, we do not propose CBTi‐seq as a substitute for microfluidic droplet‐based single‐cell methods. Instead, they are complementary methods for solving different problems. If the aim is to generate a comprehensive single‐cell atlas of a biological sample, a higher number of cells may be the best option for producing a comprehensive cell census. When the focus is on full‐length coverage to study isoform reconstruction (AS events) and multiplexing capabilities to handle hundreds of samples (especially for rare samples such as CTCs), the CBTi‐seq is an excellent choice. Despite its ease of use and commercial prospects, we have currently only processed experiments involving dozens or hundreds of multiplexed samples at the same time due to the limitations of manual operations. With the rapid increase in the number of samples, the potential for experimenter error caused by manual operations, time, and experimental costs cannot be ignored. To solve this problem, we have attempted to combine CBTi‐seq with a liquid handing robot to establish an automated multiple‐sample library preparation workflow. Through comparative experiments between manual and automated processes, CBTi‐seq has been shown to be fully compatible with the automated library preparation workflow, with better stability and shorter processing time (Figure S14, Supporting Information). We believe that with continued optimization, CBTi‐seq truly holds promise of opening new avenues for multiplexed sequencing of thousands of samples in 96‐ or 384‐well plates, and has the capability to simultaneously analyze samples from different teams in a manner similar to the sequencing operation on the Illumina platform.

In conclusion, CBTi‐seq establishes a high‐resolution framework for multiplexed sequencing of full‐length transcriptome analysis, enabling molecular‐resolution profiling of splice variants, allelic expression, and structural variations. This approach opens new avenues to study individual cells and microregion tissues with high sensitivity, scalability, and cost‐effectiveness, which will have broad applications in biological and clinical research. We expected that the design of flexible preprocessing will be of greater value in the field of single‐cell and spatial multi‐omics technology.

## 4 Experimental Section

### Microneedle‐Based Capturing System of Mouse Brain Tissue Sections

All mice (male C57BL/6J, 7–8 weeks) in this study were purchased from Shanghai Southern Model Biotechnology Co., Ltd. The procedures involving animals were reviewed and approved by the Ethics Committee of Zhongda Hospital, Southeast University (20 210 104 005). The mouse brains were isolated from the sacrificed mice and washed with PBS, followed by embedding in optimal cutting temperature compound (OCT) and stored at −80 °C before cry sectioning. The embedded surface of fresh frozen mouse brain tissue was trimmed and sliced by using microtome, and the obtained tissue sections were deposited on polydimethylsiloxane (PDMS) membrane slides. Then the sections were fixed with 4% paraformaldehyde briefly, rinsed with H_2_O to remove OCT, and dehydrated using isopropyl alcohol for 2 min. After that the treated tissue sections were stained with hematoxylin and eosin (H&E) according to previously reported literature.^[^ ^38^ ^]^ The processed tissue sections could be directly used for microneedle sampling or stored at −80 °C freezer for further processing.

Before tissue sampling, the PCR tubes or plates were pre‐added with lysis buffer according to the above described. The home‐built microneedle capturing system, originated from the group was used for tissue micro‐sampling. The microneedle capturing system was consisted of four parts, including a 3D translation stage for position control, a system control unit, a microscopic camera system for monitoring the sampling process, and a tapered glass capillary tube for tissue capturing operation. Among this, the capillary tube was fabricated by heating and stretching a glass capillary (200 µm i.d., 360 µm o.d.) in micropipette puller instrument (P‐1000, Sutter, USA) to produce a tapered tip. The obtained tissue samples were blown into microtubes by connecting a capillary tube with a compatible syringe. Finally, the tissue samples were centrifuged at 500 g for 2 min to ensure them at the bottom of the tube and were directly analyzed or frozen at −20 °C until library preparation.

### Cell Culture and Collection

The HEK293T, human hESC H9, and mouse L929 fibroblast cells were grown in a medium compatible with each cell line and all cultured for 5 days in a 37 °C incubator with 5% CO_2_. The Rrm2‐knockdown L929 cell groups were treated in the manner described in the literature.^[^ ^39^ ^]^ Then cells were detached with trypsin treatment after the supernatant was removed. Perform cell aggregate dissociation with strong pipetting. Cells were then rinsed three times with ice‐cold phosphate buffer solution (PBS), pelleted by centrifugation at 500 ×g for 10 min at 4 °C to remove the supernatant containing excess trypsin and culture media thoroughly, and finally resuspended in PBS for subsequent experiments.

The home‐built microneedle capturing system described above was employed for picking up single cells into 8‐ or 96‐well plates containing lysis buffer. Specifically, under the optimized diameter of the capillary tip (30 µm), the conical tip of the capillary probe was controlled to be aligned with the target cells, and the target cell was sucked into the probe under capillary force. The CBTi‐seq lysis buffer consisted of 0.13 µL recombinant RNase inhibitor (40 U µL^−1^, Takara), 0.25 µL of Takara lysis buffer (10 ×), and 0.5 µL CBTi‐Oligo dT primer (10 µm), and ddH_2_O to 2.5 µL final volume. The plates were centrifuged at 1000 ×g immediately after sorting and stored at −80 °C until use.

### hESC H9 Cell RNA Extraction

The standard protocols of hESC H9 RNA extraction have been described in detail in the Supporting Information. After the extraction and preparation, the obtained RNA dry powder was resuspended in ddH_2_O, and then hESC H9 RNA stocks were measured on a Qubit fluorometer with Quant‐iT RNA Assay Kit (ThermoFisher Scientific) for dilution calculation in subsequent experiments, followed by the determination of RIN values using an Agilent electrophoresis system to assess the quality of extracted RNA. All RNA stocks were stored at −80 °C until use.

### Isolation and Capture of Adult Human Testicular Cells for scRNA‐seq and AS Profiling

The human testicular tissues from donors with OA were approved by the ethics committee of Shanghai General Hospital (2022SQ294). All patients have provided written informed consent. Testicular tissues of patients with OA were minced with sterilized scissors after washing three times with PBS, then were digested in collagenase type and trypsin at 37 °C for 30 min. The digestion process was stopped with DMEM containing 10% FBS. After centrifugation and resuspension, single testicular cells were randomly selected into the prepared lysis buffer through a mouth pipette and stored at −80 °C for further analysis.

### Combinational Barcode Tn5 Transposase Preparation

The bare Tn5 transposase used in this study was purchased from Wuhan ABclonal Biotechnology Co., Ltd, and the transposition efficiency of this in vitro was 1000‐fold higher than that of wild‐type Tn5 transposase. The sequences of ME, ME‐A_m_, and ME‐B_n_ are shown in Table S3 (Supporting Information), in which the ME sequence is the chimeric end of the Tn5 transposon, and the sequence design of ME‐A_m_ and ME‐B_n_ (5′ to 3′) is described below:

1) The sequence that forms a reverse complementary binding with the ME sequence; 2) an eight‐base barcode A_m_/B_n_ index was to label tagmented dsDNA fragments. We have listed ten different barcodes of each in this study, which can be used to label up to 100 different samples by orthogonal combination barcoding, and the barcode can be further flexibly increased according to the experimental requirements of higher throughput without other conditions changing; 3) UMI was available only for ME‐B_n_, which consists of 6‐bp concatenated base N for precise quantitative sequencing of expression levels of reads; 4) Read 1 and Read 2 were served as binding sites for sequencing primer recognition.

First, 4 µL ME‐A_m_ (20 µm), 4 µL ME (20 µm) and 2 µL annealing buffer were added into tube, vortex oscillated, centrifuged and collected at the bottom of the tube, then placed in a PCR thermocycler for hybridization to obtain Mix A_m_ product. The following program was used: 75 °C for 15 min, 60 °C for 10 min, 50 °C for 10 min, 40 °C for 10 min, 20 °C for 30 min, and 4 °C hold. The protocol of Mix B_n_ preparation is the same as that described above for Mix A_m_, with the minor differences of replacing the ME‐ A_m_ sequence with ME‐B_n_. Next, 2 µL of Mix A_m_/Mix B_n_, 1 µL Tn5 transposase (1 µg µL^−1^), 5 µL assemble buffer (5 ×) and 17 µL nuclease‐free water were mixed and incubated in a PCR thermocycler at 35 °C for 1 h to complete the assembly of Barcode Tn5 transposase. The products were named Barcode A_m_ Tn5 and Barcode B_n_ Tn5, respectively. Of note, A_m_ and B_n_ are numbered according to the choice of each barcoding.

### RT‐PCR Reaction for CBTi‐seq

Sorted single‐cell or microregion tissue samples were removed from the −80 °C storage and thawed at room temperature for 10 min to facilitate sample lysis and RNA release, then transferred to a preheated PCR thermocycler, incubated for 3 min at 72 °C and immediately placed on ice afterward. Next, 10 µL of RT‐PCR mix containing 1.2 µL dNTP mix (10 mm), 0.252 µL RNase Inhibitor (40 U µL^−1^), 0.1 µL DTT (100 mm), 0.328 µL MgCl_2_ (279 mm), 0.18 µL dCTP (100 mm), 2 µL Betaine (5 m), 0.368 µL CBTi‐TSO (50 µm), 0.172 µL of Superscript IV / Maxima H (200 U µL^−1^, ThermoFisher Scientific) and 5 µL of KAPA HiFi HotStart Ready Mix (2×, Roche) were added to synthesis full‐length dsDNA. The following RT‐PCR program was set to: 60 min at 50, 98 °C for 3 min, then 20 cycles of (98 °C for 20 s, 67 °C for 20 s, 72 °C for 6 min). The cDNA concentration was quantified using the Qubit 4.0 fluorimeter, and size distribution was assessed using capillary electrophoresis on a Fragment Analyzer system (Agilent Bioanalyzer).

For the H9 RNA extracted sample, the procedure is the same as that described above, except that the lysis buffer and lysis process were removed, and the subsequent reaction was carried out by directly adding 1 µL CBTi‐Oligo dT primer (5 µm) to RNA sample.

### Barcoded Tagmentation and CBTi‐seq Library Preparation

For single independent sample library, 1 µL unpurified amplified cDNA working dilution (5 ng µL^−1^) was transferred to a new microtube before adding tagmentation reaction mix, which contained 4 µL Tagment buffer (5×, ABclonal), 1.25 µL Barcode A_m_ Tn5, 1.25 µL Barcode B_n_ Tn5, and nuclease‐free water to 20 µL final volume. The microtube was incubated for 10 min at 55 °C, and the Tn5 was inactivated with 4 µL stop buffer (1 mg mL^−1^ of protease, 0.5 m of NaCl, and 75 mm EDTA), followed by 50 °C for 40 min and 70 °C for 20 min. After that, library amplification of the tagmented samples was performed using 25 µL PCR MIX (2×, ABclonal) and 5 µL premixed custom CBTi‐Index I7 and CBTi‐Index I5 adapters (5 µm each). PCR was cycled as follows: 72 °C for 3 min, 98 °C for 30 s, and then 10 cycles of (98 °C for 15 s, 61 °C for 30 s, 72 °C for 3 min), 72 °C for 5 min, 4 °C hold. Finally, the resulting library was purified with a 0.75:1 ratio of AMPure XP beads to DNA and quantified by Qubit 4.0.

For multiplexed sample libraries, 1 µL unpurified amplified cDNA working dilution (5 ng µL^−1^) of each sample was transferred to a new plate before adding tagmentation reaction mix as described above. Of note, each sample used a unique combination of barcoding. Samples were incubated for 10 min at 55 °C. Then, all tagmented products were mixed into 1.5‐ or 2 mL PCR tube, purified with 1.5× AMPure XP beads for termination of the tagmented reaction and collection, followed by elution with 20 µL nuclease‐free water. The amplification and purification of the sequencing library were carried out as described above.

### Sequencing and Data Preprocessing

In this experiment, the sequencing library was processed using the Illumina Novaseq 6000 with a 150 bp paired‐end sequencing strategy, and the raw data consisted of two parts: Read 1.fastq.gz and Read 2.fastq.gz. The sequencing data was processed using the SSH (Secure Shell) client MobaXterm within a Linux operating system. The overall processing workflow was performed according to the following:

- Quality control: FastQC was used to assess the quality of the sequencing data fastq.gz format. Indicators such as “Per base sequence quality,” “Per base sequence content,” and “Sequence Duplication Levels” were used to make a preliminary judgment of the quality of the sequencing library.
- Barcode B and UMI swapping: The fastq.gz files were decompressed using the zcat command to convert them into “fq” files. Following the process shown in Figure S15 (Supporting Information), a Python script was written to swap the Barcode B and UMI from Read 2 to the beginning of Read 1. Additionally, 33 bp from the 5′ end of Read 2 was deleted. The Final_R1/R2.fq.gz files were then compressed.
- Data preprocessing: The FastqToSam function in Picard tools were invoked to convert the “fq” file into query‐name sorted bam file. Run the TagBamWithReadSequenceExtended function from Drop‐seq_tools‐3.0.2‐0 twice to extract Barcode and UMI sequences at different locations, then invoke the FilterBam function to filter low‐quality Barcode and UMI sequences, and finally use the PolyATrimmer function to remove the Poly A sequence at the capture end. After preprocessing, the SamToFastq function from Picard tools was used to convert the bam files back into fastq files for subsequent sequence alignment.
- Sequence alignment: Reference genome indices for mouse GRCm38 (https://www.ncbi.nlm.nih.gov/grc/mouse) and human GRCh38 (https://www.ncbi.nlm.nih.gov/grc/human) were constructed from NCBI Genome Reference Consortium, respectively. Sequence alignment was then performed using STAR software (version 2.7.11b).
- Expression matrix: After alignment, the SortSam and MergeBamAlignment functions in Picard were called to sort and integrate the results. Finally, the DigitalExpression function in Drop‐seq_tools‐3.0.2‐0 was employed with specified filtering conditions to select the digital expression matrix corresponding to the specified Barcode.

A complete description of the materials, sequences, and methods is provided in Supporting Information.

### Bioinformatics Analysis

The HEK293T single‐cell gene expression matrix data of smart‐seq2/3 and FLASH‐seq were obtained from the literature.^[^ ^21^ ^]^ The raw read counts were summarized to normalize and log‐transformed gene expression for further analysis using DESeq2. Coverage across the gene body was calculated by RseQC. Reads Coverage and Sashimi plot of sequencing data aligned at the Rrm2 locus was visualized using the integrated genome viewer (IGV, version 2.17.4) genome browser. Gene ontology analysis of enriched gene sets in the most variable 2000 genes was carried out in iDEP,^[^ ^40^ ^]^ and the enriched terms with adjusted p values less than 0.01. The splicing patterns profiling in adult human testicular cells were analyzed by Marvel package. The extracted data were further analyzed and visualized using Origin, online analysis tool (https://cloud.oebiotech.cn) and R package (version 4.3.2).

## Conflict of Interest

The authors declare no conflict of interest.

## Supporting information



Supporting Information

## Data Availability

The data that support the findings of this study are available from the corresponding author upon reasonable request.
