# Supplementary material for: High‐Resolution Multiplexed Sequencing of Single‐Cell Full‐length Transcriptome Via Combinational Barcoded Tn5 Transposon Insertion
Source: Adv Sci (Weinh). 2025 Nov 11;13(1):e16013. doi: 10.1002/advs.202516013 (PMC12766991; doi:10.1002/advs.202516013)
Supplement: Supplementary file 1 — Supporting Information [file ADVS-13-e16013-s001.docx]

**Supplementary material**

**High-resolution Multiplexed Sequencing of Single-cell Full-length Transcriptome via Combinational Barcoded Tn5 Transposon Insertion**

Liyong He^1, #^, Kaitong Dang^1, #^, Qian Sun^2, #^, Wenjia Wang^1^, Wenbo Li^3^, Wenyi Zhang^1^, Kaiqiang Ye^1^, Handong Wang^1^, Zhengyue Li^4^, Yan Guo^5^, Zheng Li^3,6,7^, Chencheng Yao^3^, Peng Li^3^, Yan Huang^1^, Xiangwei Zhao^1, *^

^1^*State Key Laboratory of Digital Medical Engineering, School of Biological Science & Medical Engineering, Southeast University, Nanjing, 211189, China*

^2^ *State Key Laboratory of Reproductive Medicine and Offspring Health, Nanjing Medical University, Nanjing, 211166, China*

^3^*Department of Andrology, Center for Men’s Health, Department of ART, Institute of Urology, Urologic Medical Center, Shanghai Key Laboratory of Reproductive Medicine, Shanghai General Hospital, Shanghai Jiao Tong University School of Medicine, Shanghai, 200080, China*

^4^ *Feinberg School of Medicine, Northwestern University*

^5^*School of Biomedical Engineering, Shanghai Jiao Tong University, Shanghai, 200242, China*

^6^*State Key Laboratory of Reproductive Medicine and Offspring Health, Nanjing Medical University, Nanjing, 211166, China*

^7^*The Affiliated Taizhou People’s Hospital of Nanjing Medical University, Taizhou School of Clinical Medicine, Nanjing Medical University, Taizhou, 225300, China*

**^#^**These authors contributed equally

^*^Xiangwei Zhao, email: xwzhao@seu.edu.cn

**Methods**

**PDMS slide preparation.**

The PDMS membrane was cut into a rectangle of 18 mm × 24 mm, the protective film was removed from one side of the membrane, and the slides were placed in the cleaning chamber of the plasma machine together with the slides, the valve of the oxygen cylinder of the cleaning gas line was opened, and the vacuum pump was turned on to evacuate the cleaning chamber. The cleaning intensity was 700 V, and the cleaning time was 2 min. Open cleaning machine, and the plasma generated by oxygen ionization was used to clean the surfaces of the PDMS membrane and the slides, and then the PDMS membrane and the slides were aligned to fit together, and the surface of the membrane was gently pressed by tweezers to complete the irreversible bonding.

**H9 embryonic stem cell RNA extraction.**

Take T25 culture flasks, wash with PBS for 3 times, add 3 mL Trizol to each flask and stand at room temperature for 5 min, use a pipette gun to blow down the adherent cells and transfer to 1.5 ml centrifuge tubes, and lysis at room temperature for 25 min. Add 1/5 volume of chloroform (150 μl per tube), vortex on the mini-mixer for 15 s, stand for 20 min at room temperature, centrifuge at 12000 rpm for 15 min at 4℃, and put the supernatant into 1.5 mL centrifuge tubes. Add an equal volume of isopropanol (~750 μL), turn up and down 5-7 times, let stand for 45 min, and then centrifuge at 4℃ 12000 rpm for 10 min (white precipitate at the bottom can be observed). Discard the supernatant, add 1 mL of pre-cooled 75% ethanol, gently up and down to wash the centrifuge tube, 4 ℃ 12000 rpm, centrifugation for 15 min, discard ethanol, ultra-clean table fan drying to the centrifuge tube of the white material in the semi-transparent (in which the white material is the RNA). An appropriate amount of enzyme-free water (depending on the amount of RNA extracted) was added to dissolve and centrifuge for 5 min.

**Table S1. Comparative experimental design of stop buffers**

| **Exp.ID** | **Stop buffer** | **Volume(µl)** | **Procedure** | **Ref** |
| --- | --- | --- | --- | --- |
| S-1 | 50 mM EDTA | 1.05 | 68℃, 30 min | LIANTI |
| S-2 | Protease (1 mg/ml) +NaCl (0.5 M) +EDTA (75 mM) | 4 | 50℃, 40 min; 70℃, 20min | SHERRY |
| S-3 | 1.5×AMPure XP beads | 30 | Purify | Vazyme S601 Kit |
| S-4 | N/A | N/A | N/A | N/A |

**Table S2. Comparison of performance and cost among Smart-seq2, Smart-seq3 and FLASH-seq.**

|  | **Smart-seq2** | **Smart-seq3** | **FLASH-seq** | **CBTi-seq**  **(This work)** |
| --- | --- | --- | --- | --- |
| **Sensitivity of gene detection** (e.g. HEK293T cell, 500K raw reads) | ~8,000 | ~10,000 | ~11,000 | **~11,300** |
| **Workflow time** | ~10 h | ~9 h | ~4.5-7 h | **~5 h** |
| **Gene-level quantification** | No | Yes | Yes | **Yes** |
| **Transcript-level quantification** | No | No | No | **Yes** |
| **Multiplexed library construction** | No | No | No | **Yes** |
| **Cost**  (96 single cells) | ~4,320 $ | ~111 $ | ~96 $ | **~72 $** |
| **Cost**  (384 single cells) | ~17,280 $ | ~445 $ | ~384 $ | **~286 $** |

Note: The data regarding the sensitivity of gene detection among Smart-seq2, Smart-seq3 and FLASH-seq are derived from the experimental results reported in the FLASH-seq study.

**Table S3. The sequence oligonucleotides used in this strategy.**

| **Name** | **Sequence (5'—3′)** |
| --- | --- |
| **ME** | 5'-phos-CTGTCTCTTATACACATCT-NH_2_-3' |
| **ME-A_m_** | 5'-ACACTCTTTCCCTACACGACGCTCTTCCGATCT [Barcode A] AGATGTGTATAAGAGACAG-3' |
| **ME-B_n_** | 5'-GTGACTGGAGTTCAGACGTGTGCTCTTCCGATCT [Barcode B] NNNNNNAGATGTGTATAAGAGACAG-3' |
| SS2-Oligo dT | 5′-AAGCAGTGGTATCAACGCAGAGTACTTTTTTTTTTTTTTTTTTTT  TTTTTTTTTTVN-3′ |
| SS2-TSO | 5'-AAGCAGTGGTATCAACGCAGAGTACATrGrGG-3' |
| ISPCR | 5′-AAGCAGTGGTATCAACGCAGAGT-3′ |
| **CBTi-Oligo dT** | 5′-Bio-AAGCAGTGGTATCAACGCAGAGTACTTTTTTTTTTTTTTTTT  TTTTTTTTTTTTTTVN-3′ |
| **CBTi-TSO** | 5′-Bio-AAGCAGTGGTATCAACGCAGAGTACrGrGrG-3′ |
| SS2-Index I5 | 5'-AATGATACGGCGACCACCGAGATCTACAC[i5]TCGTCGGCAGC  GTC-3' |
| SS2-Index I7 | 5'-CAAGCAGAAGACGGCATACGAGAT[i7]GTCTCGTGGGCTCGG-3' |
| **CBTi-Index I5** | 5'-AATGATACGGCGACCACCGAGATCTACAC[i5]ACACTCTTTCCC  TACACGAC-3' |
| **CBTi-Index I7** | 5'-CAAGCAGAAGACGGCATACGAGAT[i7]GTGACTGGAGTTCAGA  CGTGT-3' |
| 3'Target-Oligo dT | 5'-TCGTCGGCAGCGTCAGATGTGTATAAGAGACAG [3’ Barcode] NNNNNNTTTTTTTTTTTTTTTTTTTTTTTTTTTTTTVN-3' |
| 3'Target-TSO | 5'-TCGTCGGCAGCGTCAGATGTGTATAAGAGArGrGG-3' |
| 3'PCR Primer | 5′-TCGTCGGCAGCGTCAGATGTGTATAAGAGA-3′ |

SS2: Smart-seq2;

Note: bold font represents the primer sequences involved in CBTi-seq method. The underlined part represents the complementary sequence of ME.

Barcode A_m_ and Barcode B_n_ sequence lists:

| **Barcode A_m_ ID** | **Sequence** | **Barcode B_n_ ID** | **Sequence** |
| --- | --- | --- | --- |
| Barcode A-1 | TATAGCCT | Barcode B-1 | CGAGTAAT |
| Barcode A-2 | ATAGAGGC | Barcode B-2 | TCTCCGGA |
| Barcode A-3 | CCTATCCT | Barcode B-3 | AATGAGCG |
| Barcode A-4 | GGCTCTGA | Barcode B-4 | GGAATCTC |
| Barcode A-5 | AGGCGAAG | Barcode B-5 | TTCTGAAT |
| Barcode A-6 | TAATCTTA | Barcode B-6 | ACGAATTC |
| Barcode A-7 | CAGGACGT | Barcode B-7 | AGCTTCAG |
| Barcode A-8 | GTACTGAC | Barcode B-8 | GCGCATTA |
| Barcode A-9 | GCGCGAGA | Barcode B-9 | CATAGCCG |
| Barcode A-10 | CTATCGCT | Barcode B-10 | TTCGCGGA |
| Barcode A-**…** | **…** | Barcode B-**…** | **…** |

3' Barcode sequence in 3' Targeting kit lists:

| **Barcode ID** | **Sequence** | **Barcode ID** | **Sequence** |
| --- | --- | --- | --- |
| Barcode-1 | TGCATACC | Barcode-11 | TGACAACA |
| Barcode-2 | CATTTGTG | Barcode-12 | GAAGCGTC |
| Barcode-3 | GCGAGACT | Barcode-13 | CCTTTGGC |
| Barcode-4 | ACTACGGC | Barcode-14 | ACTCTCTT |
| Barcode-5 | GCTAGGGC | Barcode-15 | AACTTTTA |
| Barcode-6 | GGCTTATG | Barcode-16 | CCTAGGGC |
| Barcode-7 | TAGACTTG | Barcode-17 | GACCTACC |
| Barcode-8 | GTTCAGCC | Barcode-18 | GGGTTGTG |
| Barcode-9 | ACACAACT | Barcode-19 | TTTCTACC |
| Barcode-10 | CTCCCTTA | Barcode-20 | CCTAAGCG |


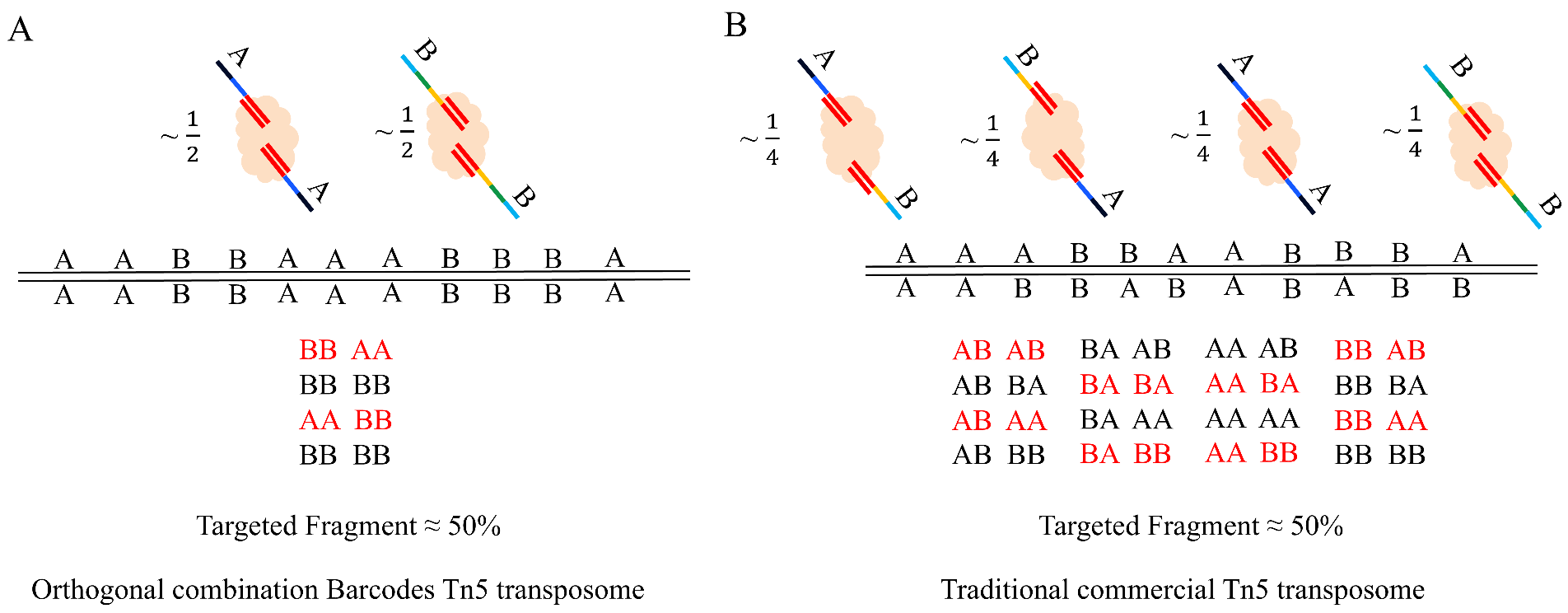


**Figure S1. Comparison of the tagmentation efficiency between CBTi-seq and commercial Tn5 transposase products.** (A) The probability of successful fragmentation on the orthogonal combination Barcode Tn5 transposome by CBTi-seq. (B) The probability of successful fragmentation on the traditional commercial Tn5 transposome.


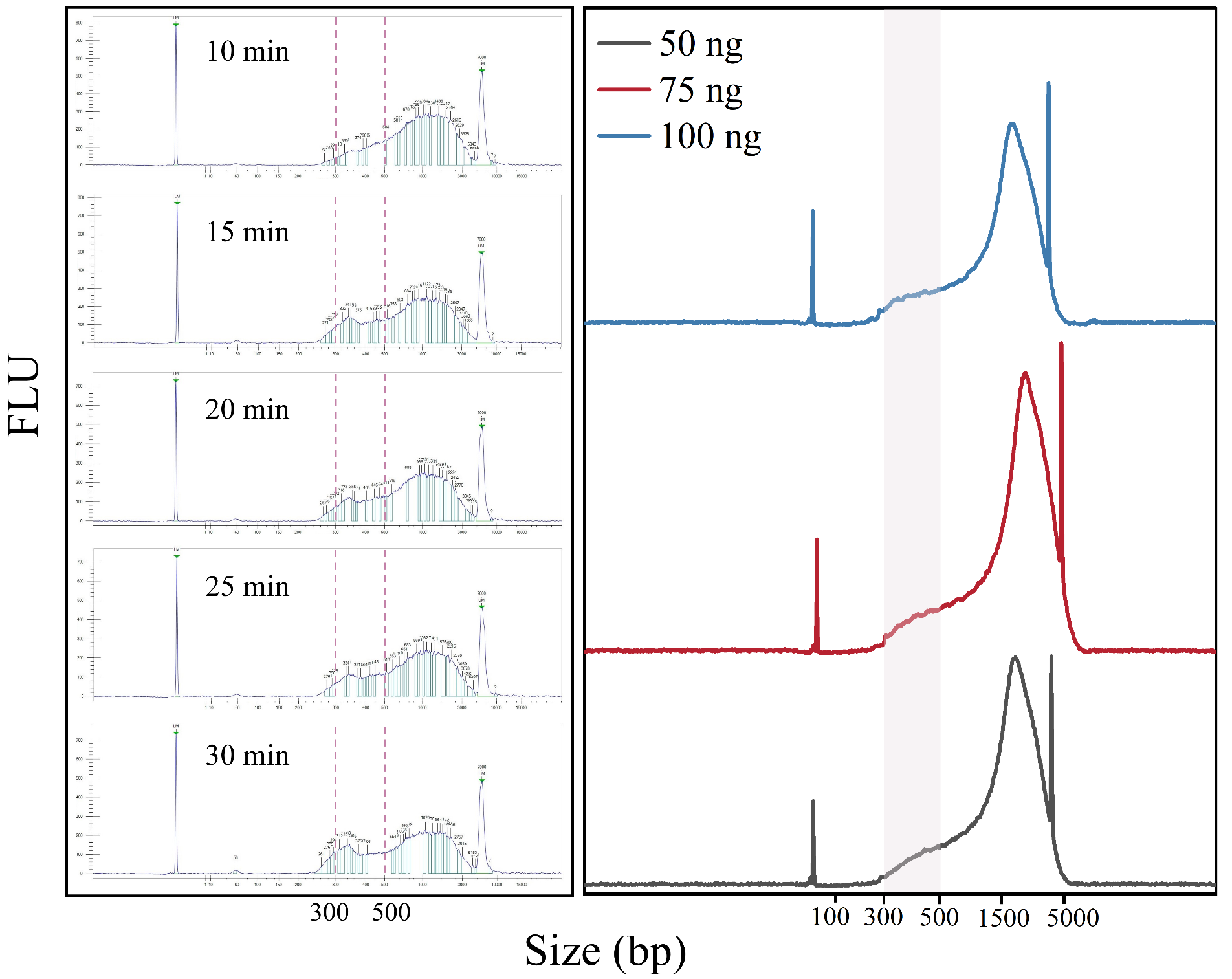


**Figure S2. The effect of different tagmentation time or input of dsDNA on the size distribution of dsDNA fragmentation products (300-500 bp).** Left panel: 10-30 min of tagmentation time. Right panel: 50-100 ng of dsDNA input.


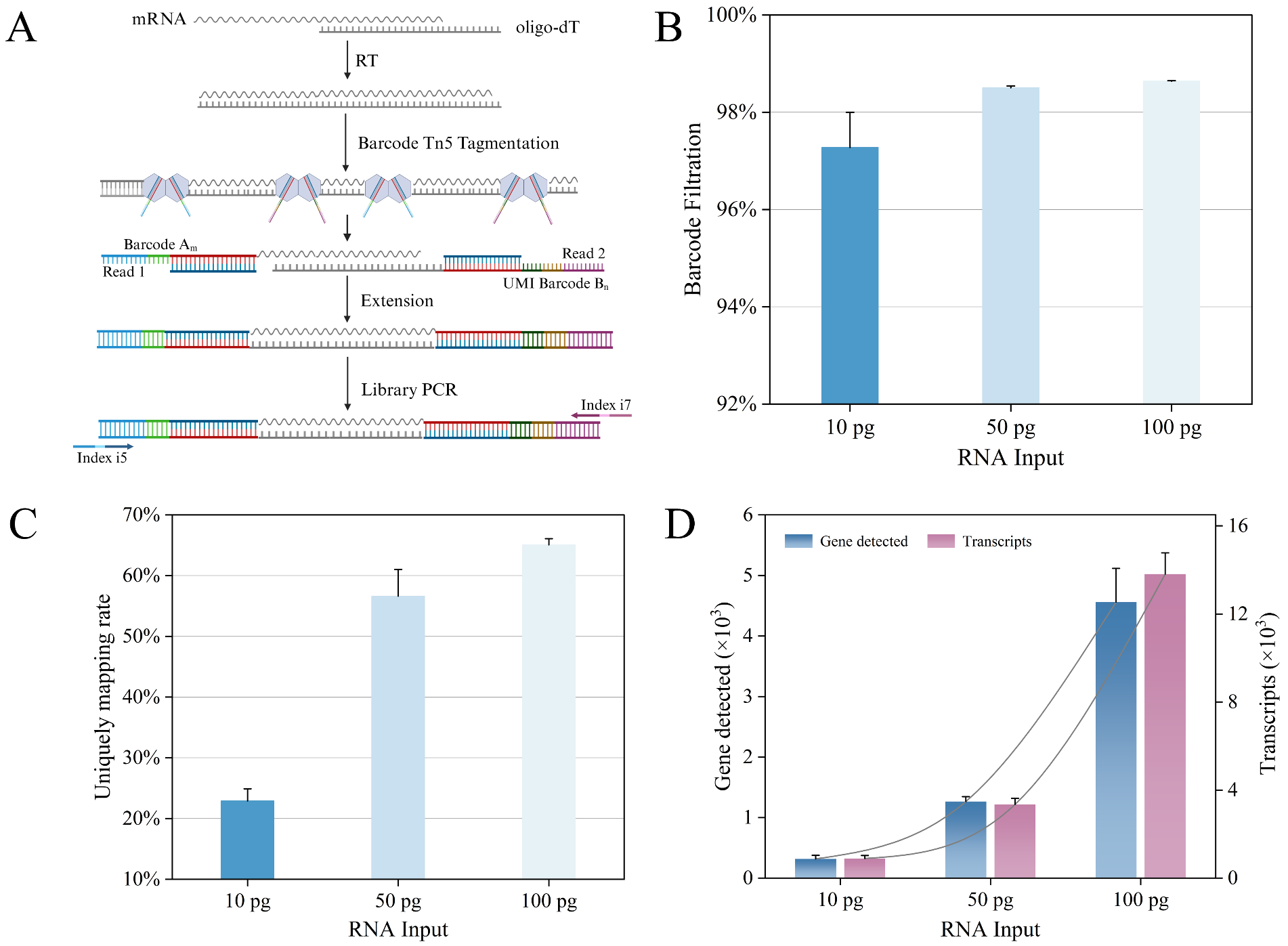


**Figure S3. Workflow and performance of direct tagmentation of RNA/DNA hybridization with Barcode Tn5 transposome for RNA-seq.** All experiments used H9 total RNA as input.

(A). Overview of CBTi-seq direct tagmented mRNA/DNA hybrids workflow. (B) Barcode filtration, (C) uniquely mapping rate and (D) gene & transcript detected from triplicate analysis of 10, 50, and 100 pg RNA input (n=3).


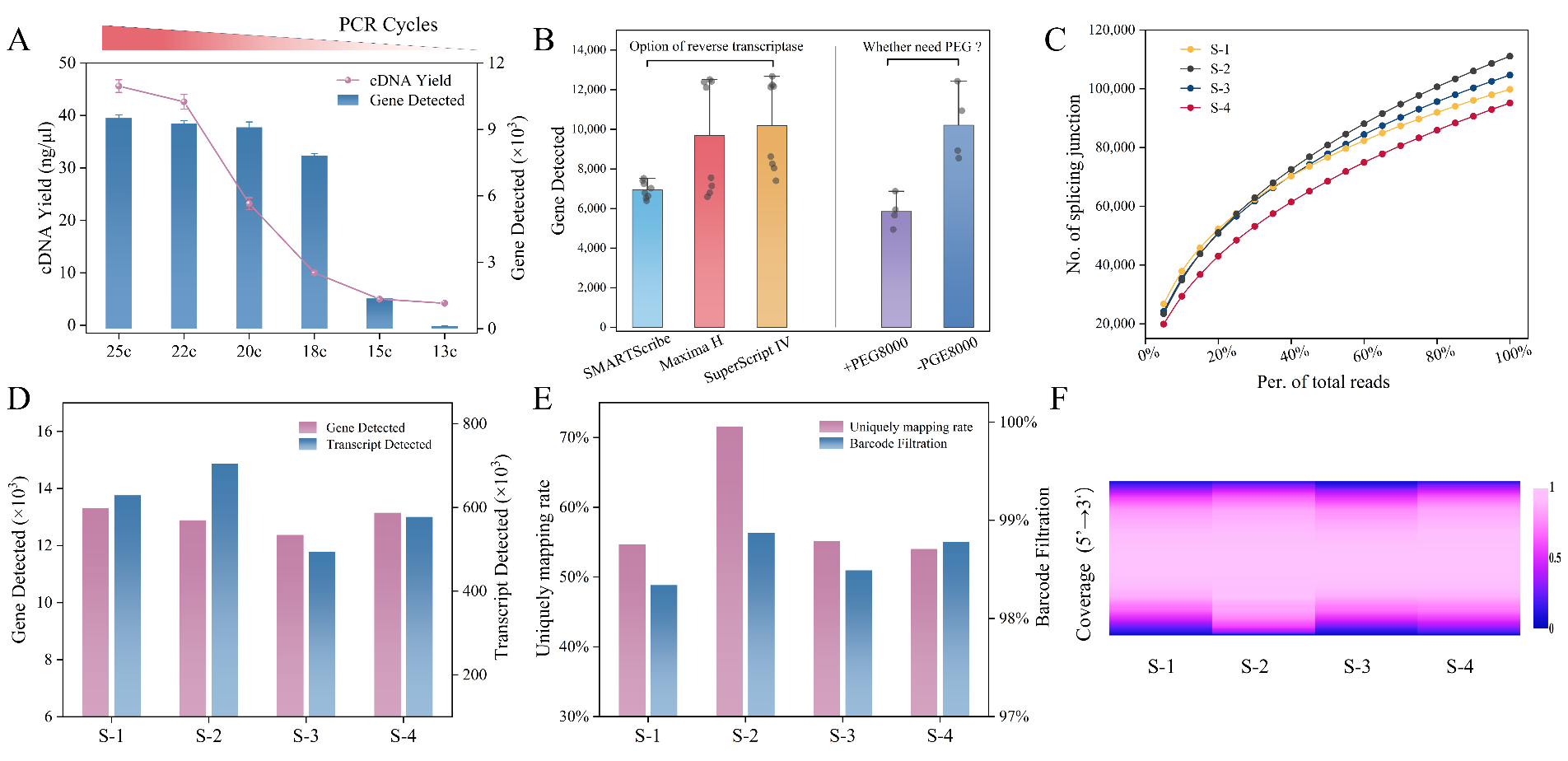


**Figure S4. Optimization of CBTi-seq and performance. All experiments used H9 total RNA as input.**

(A). Impact of the number of PCR cycles (15-25 cycles) used for cDNA preamplification on cDNA yield, in ng/µl and number of gene detected (n=3).

(B). Number of genes under various reverse transcriptase (left) detected by CBTi-seq and whether PEG8000 was added to the RT-PCR step (right) (n=3).

(C-F). Optimal stop Buffer of Tn5 transposase termination reaction with four different comparative groups. The number of splicing junctions across different downsampling thresholds (C), the number of detected annotated genes and transcripts (D), uniquely mapping rate and barcode filtration (E), and gene body coverage heatmap (5’→3’) for each stop buffer (F).


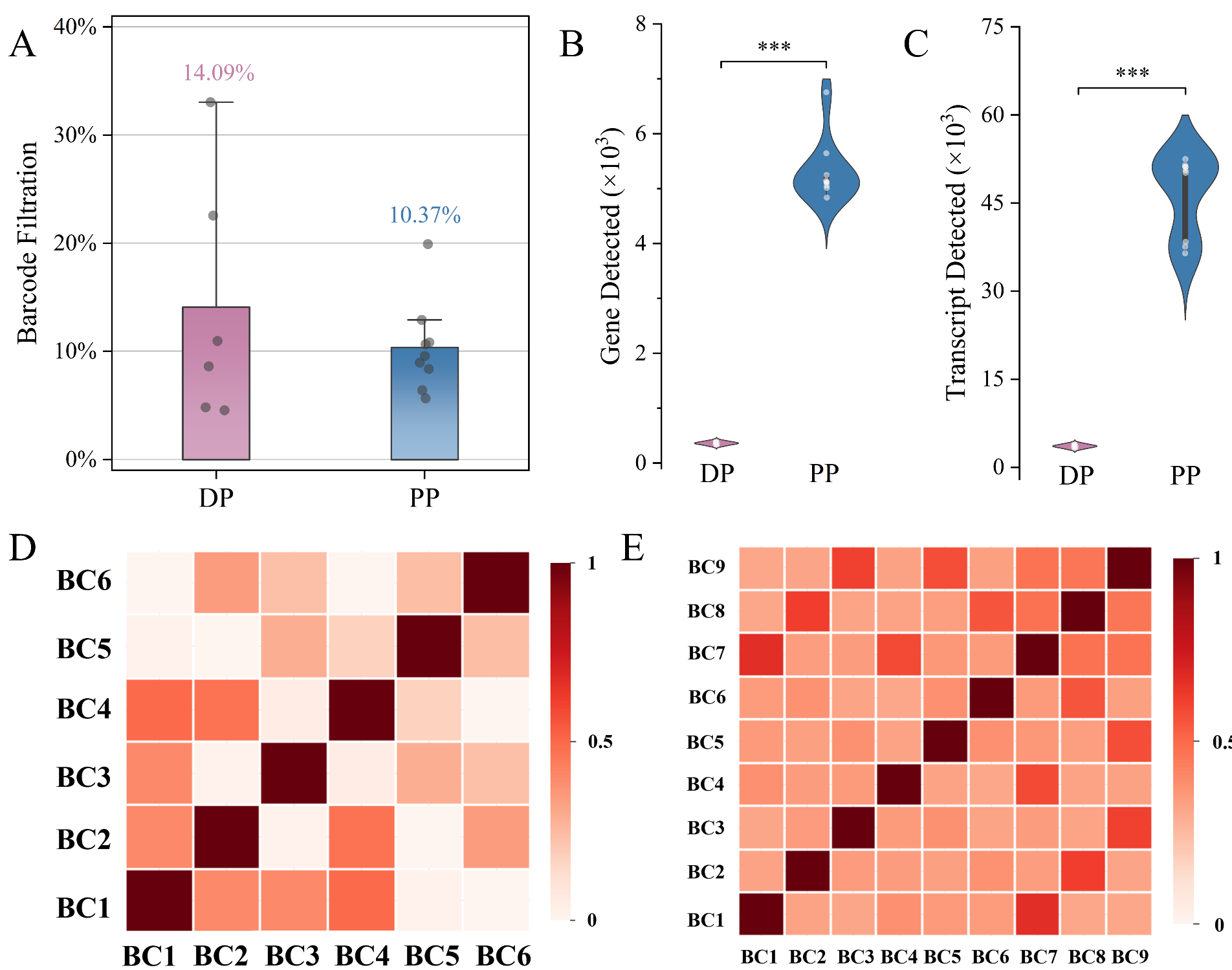


**Figure S5. Comparison of collecting mixed samples between direct pooling (DP, n=6) and purification pooling (PP, n=9).** All experiments used micro-region mouse brain tissues at single-cell resolution based on microneedle sampling system.

(A). The proportion of barcode filtration of each sample based on DP or PP collection methods. On average, 14.09 ± 10.3% barcode filtration by DP and 10.37 ± 3.9% by PP, data represent mean ± SD. Corresponding to the theory, the barcode filtration corresponding to DP of each sample and PP of each sample should be 1/6 = 16.7% and 1/9 = 11.1%, respectively.

(B). Comparison of the gene numbers using DP and PP. An average of 360 (median = 361, n=6) and 3608 (median = 3610, n = 9) gene numbers were identified in DP and PP.

(C). Comparison of the transcripts using DP and PP. An average of 5325 (median = 5114, n=6) and 46579 (median = 50560, n = 9) transcripts were identified in DP and PP. *p < 0.05, **p < 0.01, ***p < 0.001.

(D). Pearson’s correlation heatmap under DP for six samples.

(E). Pearson’s correlation heatmap under DP for nine samples.


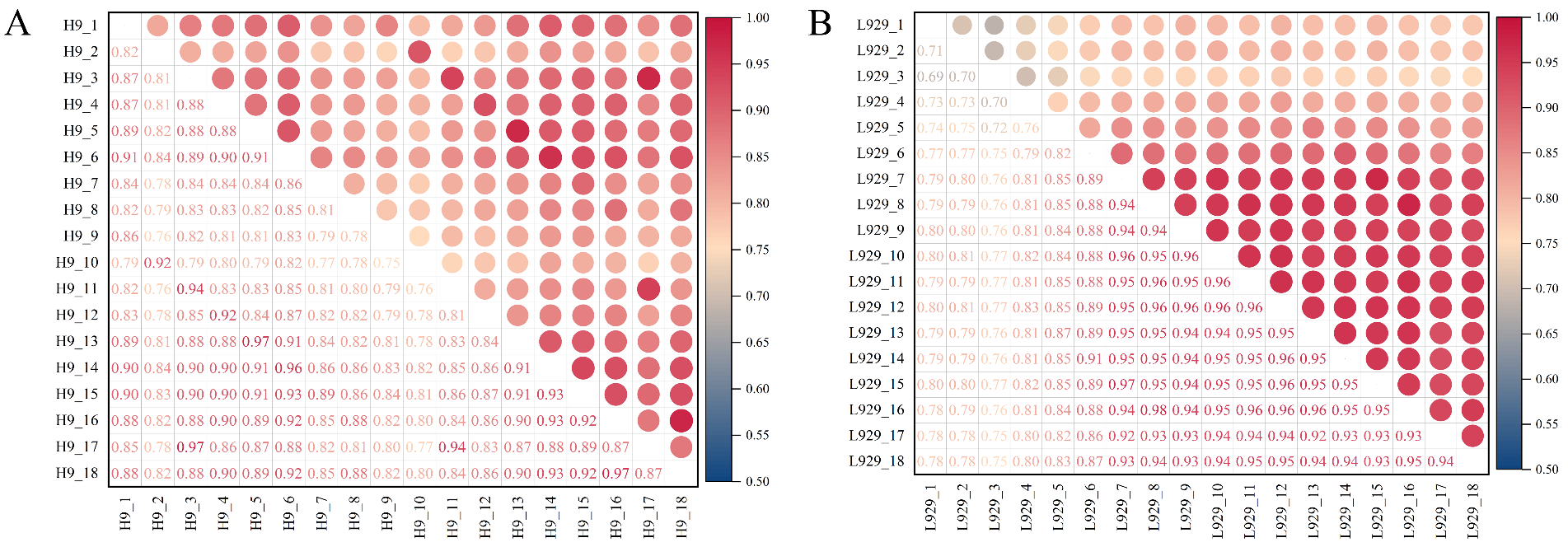


**Figure S6. Heatmap of Pearson correlation coefficients showing the reproducibility among different types of cells.** (A) L929 cells, an average of 0.865 (n=18). (B) H9 cells, an average of 0.852 (n=18).


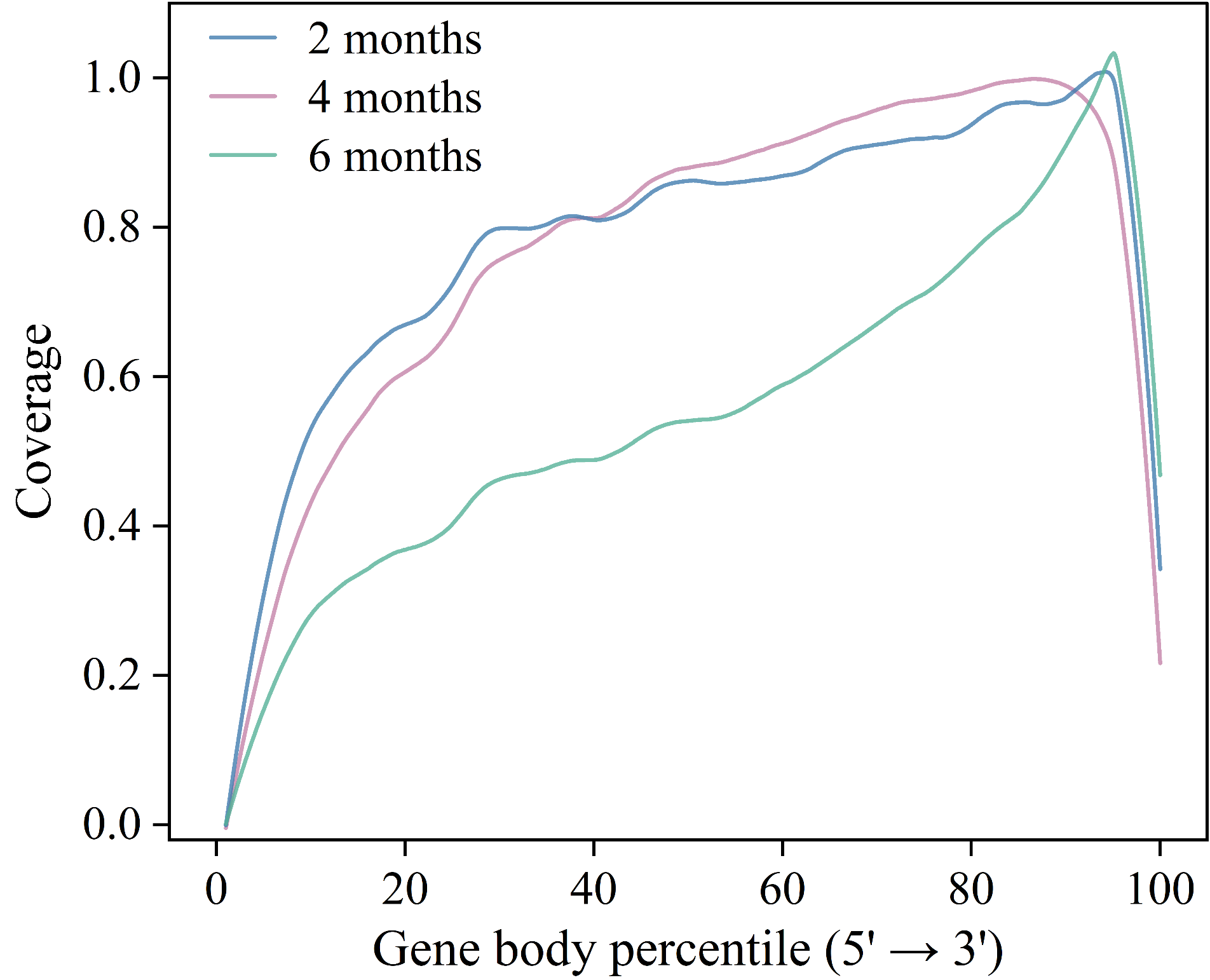


**Figure S7. The sequencing gene body coverage ability of frozen mouse brain tissue samples under different storage times (2 months, 4 months, and 6 months).**


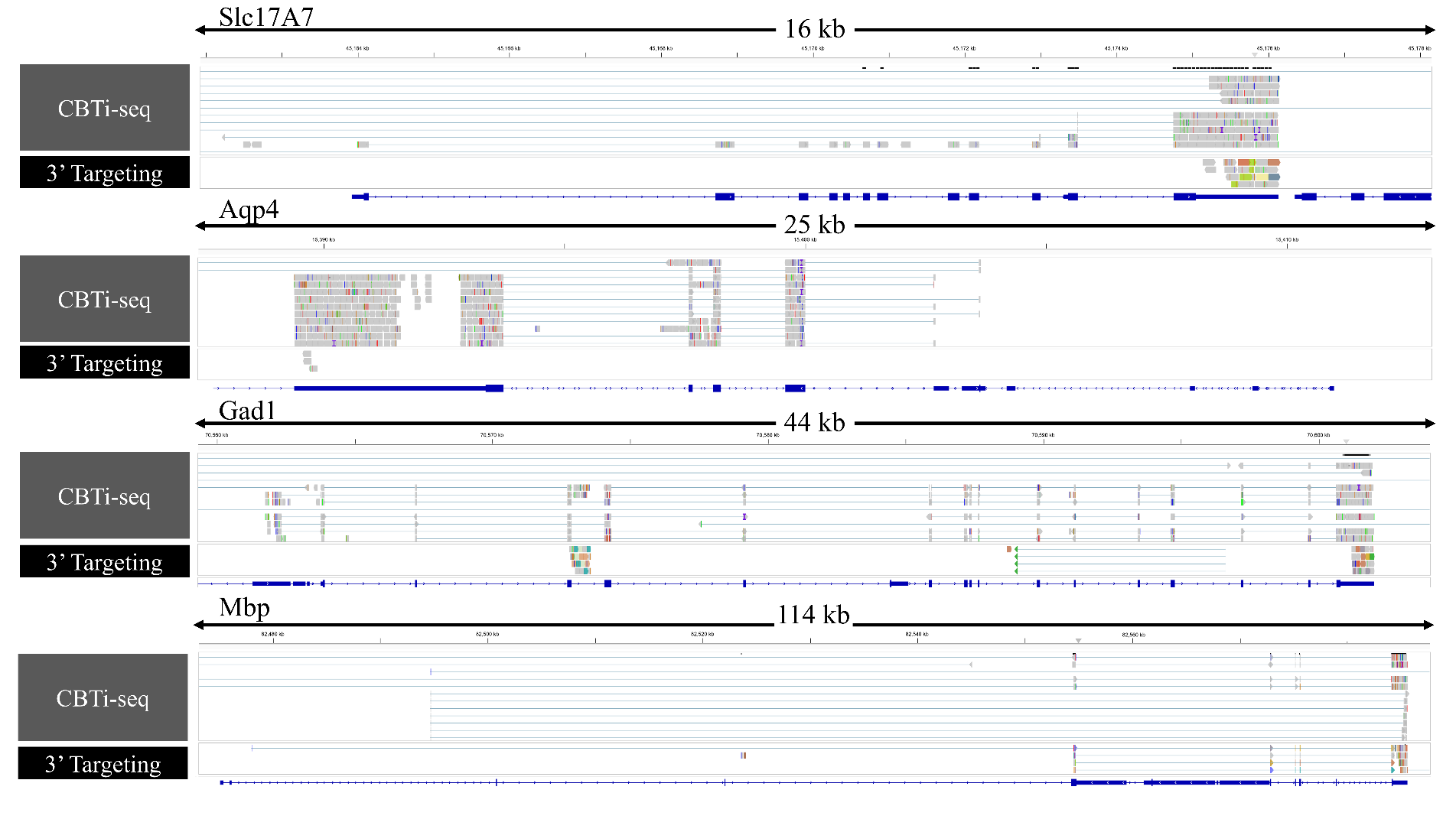


**Figure S8. Integrated Genome Viewer (IGV) visualization of selected genes from mouse brain tissues processed with CBTi-seq and 3’ Targeting kit.** In the read mapping track, each bar corresponds to a single mapped read. The color of the bar indicates the read orientation compared to the reference. Fine lines highlight split reads. The gene annotation is displayed below each panel. Fine blue lines correspond to introns and bold blue lines to exons. Each panel shows a different representative gene (from top to bottom: Slc17A7, Aqp4, Gad1 and Mbp).

**
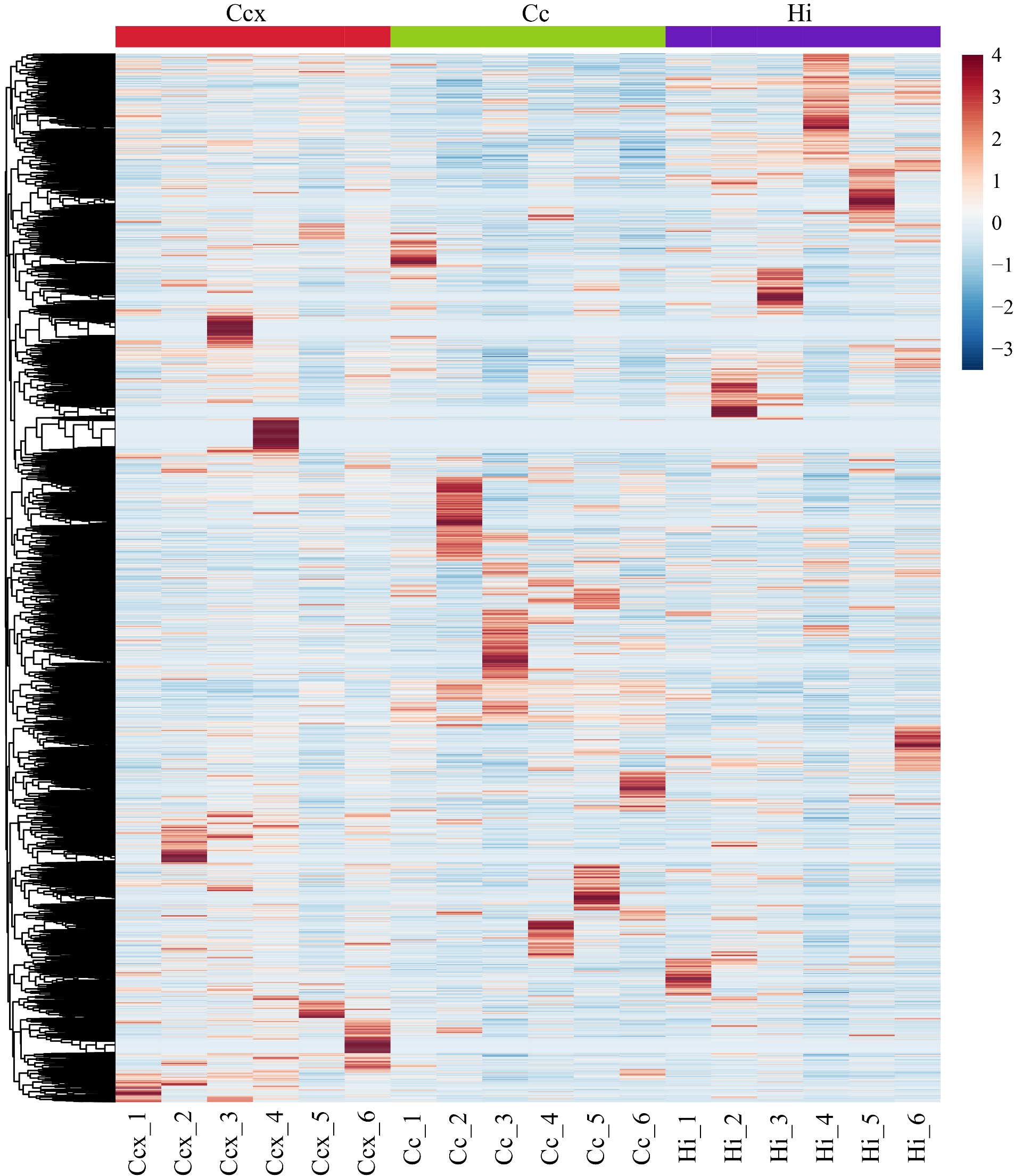
**

**Figure S9. Hierarchical cluster analysis (HCA) segregated the three brain tissue regions and differentially expressed genes.** Red: up-regulated; Blue: down-regulated.

**
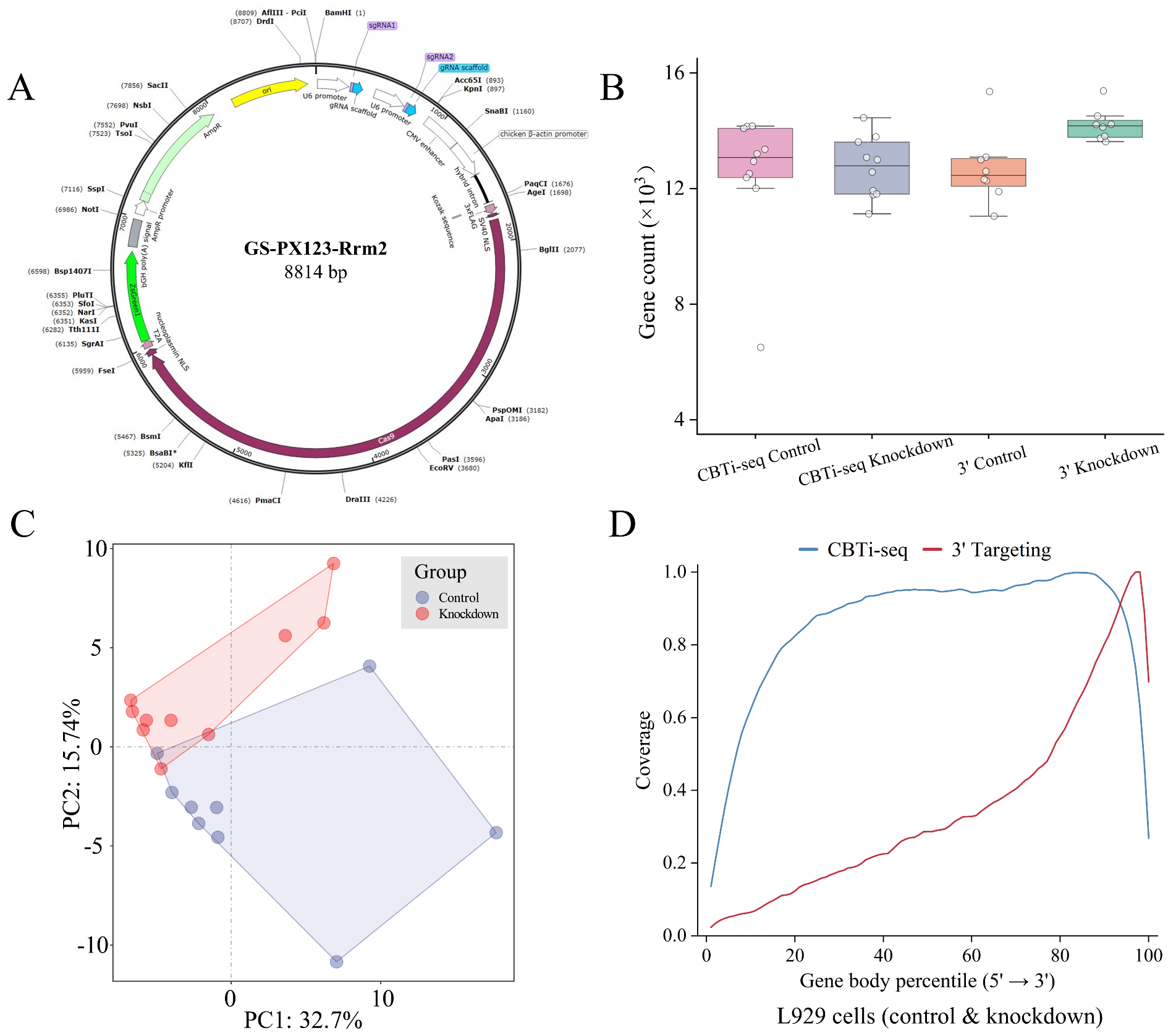
**

**Figure S10.** (A) The plasmid vector construction model for knockout of the Rrm2 genome. (B) The number of detected genes between knockdown and control L929 cell groups using CBTi-seq and 3’ targeting methods (n=10 for each group). (C) Principal component analysis of single-cell transcriptomic data between knockdown and control groups by CBTi-seq. (D) Gene body coverage with CBTi-seq and 3’ Targeting method showing the differences in sequencing coverage of CBTi-seq and 3’ Targeting (n=20 for each method).

**
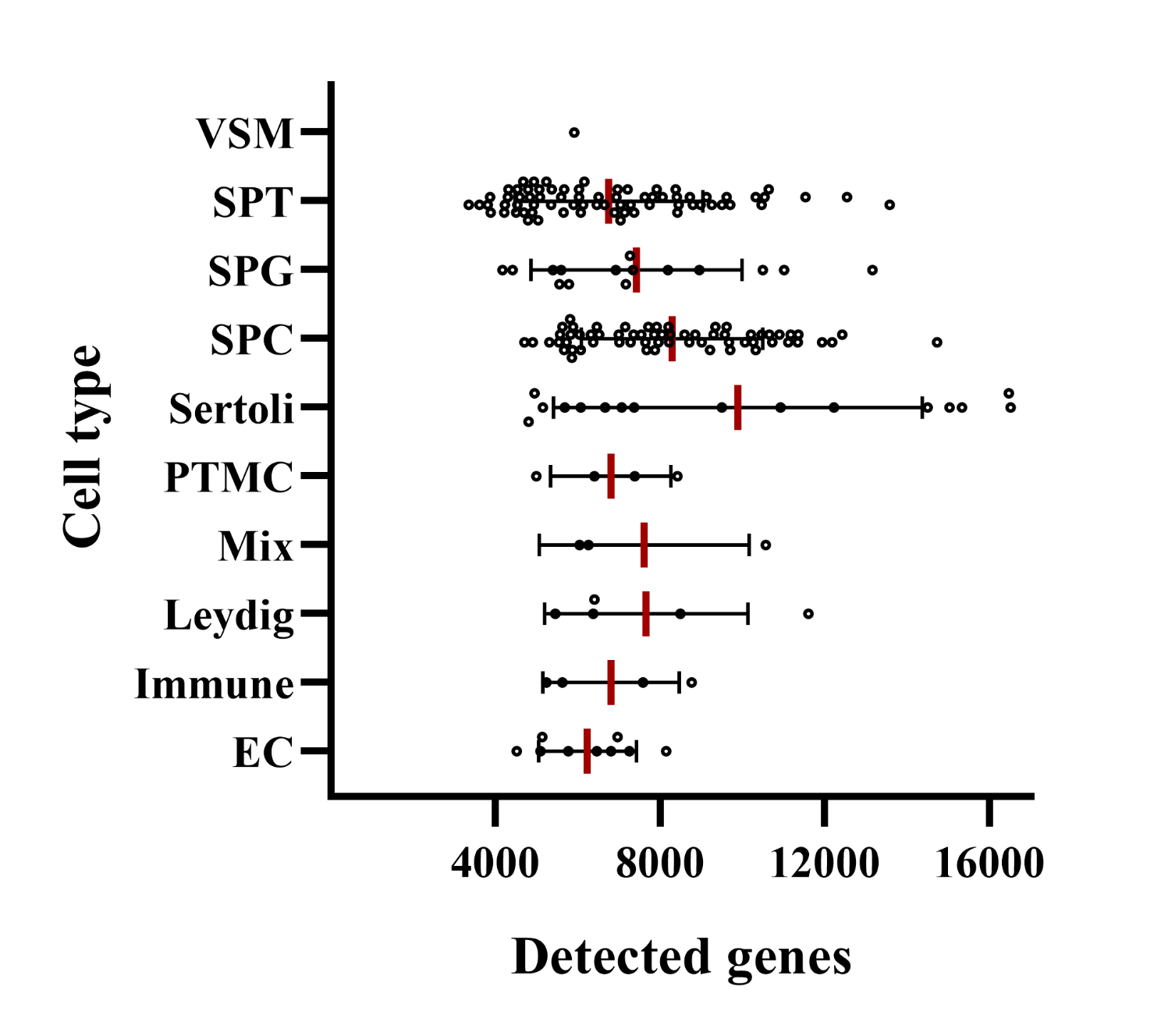
**

**Figure S11. The average gene identification results from samples of different cell types derived from testicular cells (n=192).** The red line represents the mean value.

**
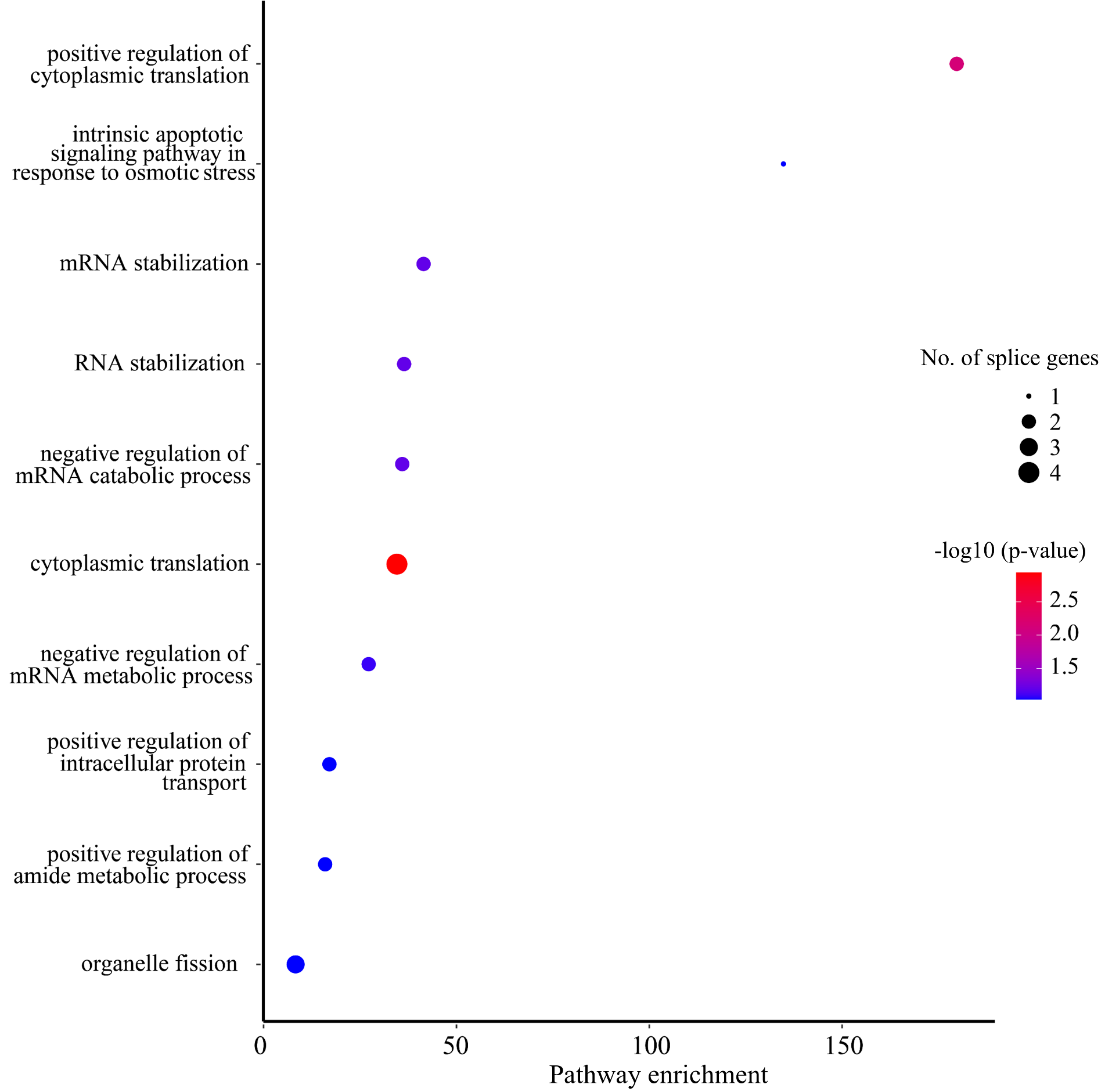
**

**Figure S12. Enrichment scores, p-values, and gene set sizes of selected biological pathways enriched among differentially spliced genes in SPC and SPG cells.**

**
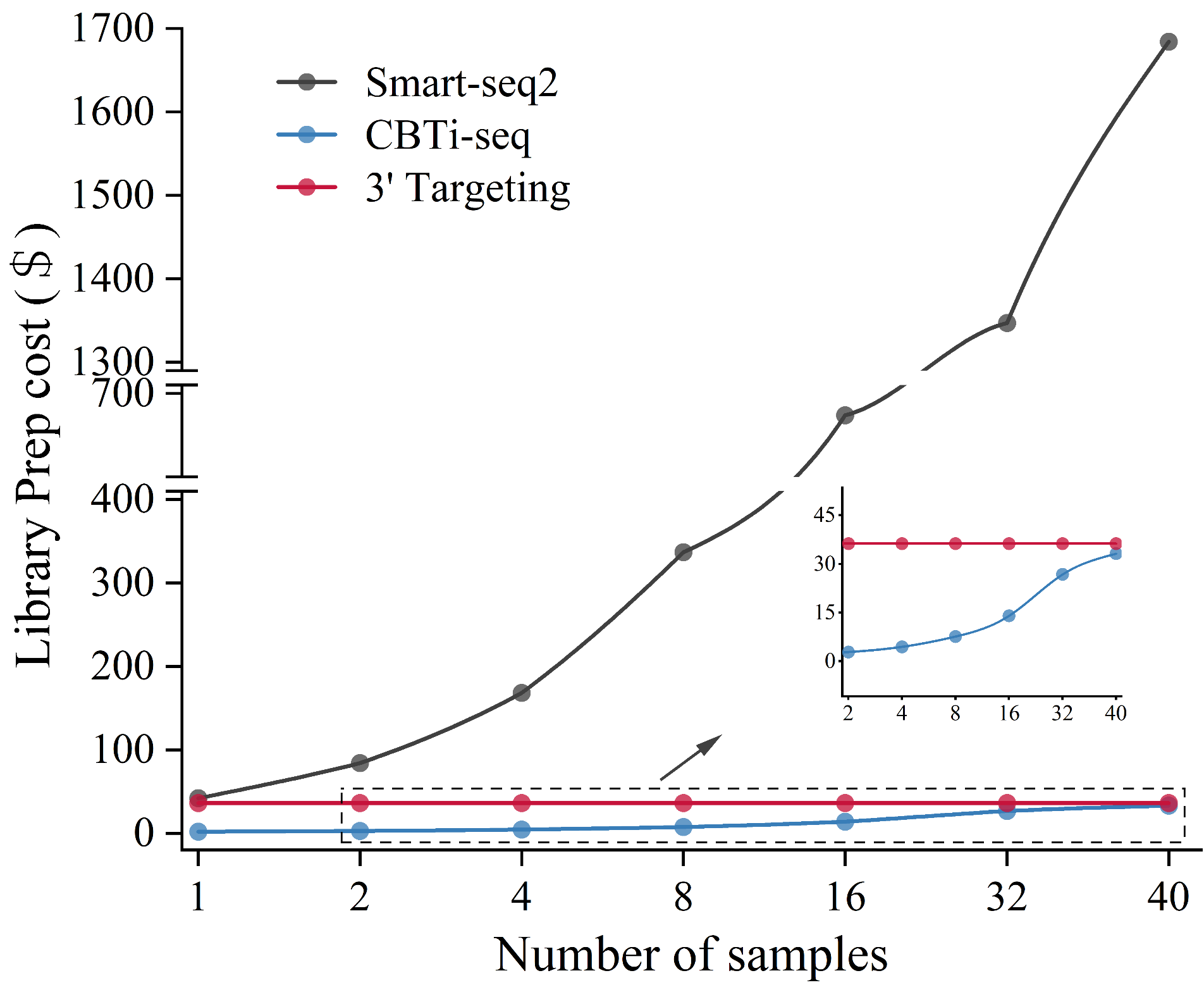
**

**Figure S13. The relationship between the cost of CBTi-seq, Smart-seq2, and 3’ Targeting kit and the increase in the number of samples.**


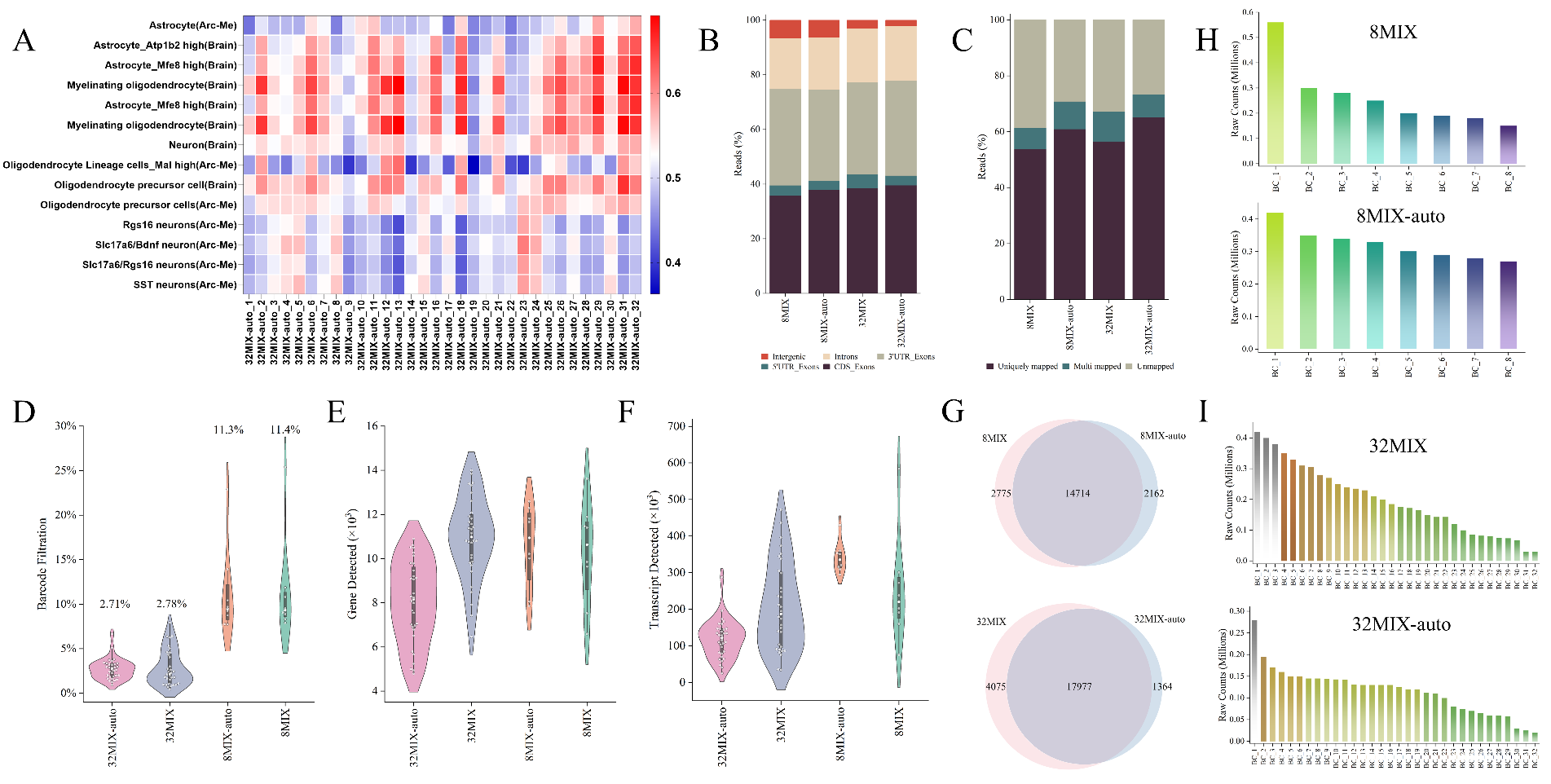


**Figure S14. Comparison of CBTi-seq workflow between manual operation and liquid handing robot-based automatic operation.** Both methods used single-cell resolved mouse brain tissues as input, with 8-MIX (auto) and 32-MIX (auto) multiplexed analysis.

(A). Pearson’s correlation heatmap for cell type identification under 32-MIX_auto. Common astrocytes, myelin cells, oligodendrocytes, excitatory neurons (Slc17a6 neuron) and heterogeneous neurons (SST neuron) in mouse brain were identified between samples.

(B). Bar plots showing the percentage of read tags mapped to CDS exons, 5’UTR_Exons, 3’UTR_Exons, intronic or intergenic features processed with 8MIX (manual or auto) and 32MIX (manual or auto), measured using ReSQC.

(C). Bar plots showing mapping statistics with the percentage of uniquely mapped, multi-mapped or unmapped reads for 8MIX (manual or auto) and 32MIX (manual or auto).

(D). The proportion of barcode filtration of each sample under different operation. On average, (mean ± SD) 2.71 ± 1.1%, 2.78 ± 2.0%, 11.3 ± 5.1%, and 11.4 ± 5.7% barcode filtration by 32MIX_auto, 32MIX, 8MIX_auto, and 8MIX, respectively. Corresponding to the theory, the barcode filtration corresponding to 32MIX of each sample and 8MIX of each sample should be 1/32 = 3.1% and 1/8 = 12.5%, respectively.

(E). Number of genes detected in 8 and 32 multiplexed samples processed with manual or automatic. Distribution of values is shown as a violin plot with a dot indicating the median.

(F). Number of transcripts detected in 8 and 32 multiplexed samples processed with manual or automatic. Distribution of values is shown as a violin plot with a dot indicating the median.

(G). Venn diagram of the common identified genes in 8MIX (top panel) or 32MIX (bottom panel) samples under manual and automatic operation. The results showed that there was no significant difference between the identification depth of the manual and automatic operation.

(H). The proportion of raw counts for the barcode of each sample in the sequencing library. Top panel: 8MIX for manual operation; Bottom panel: 8MIX for automatic operation. The results showed that the proportion of automated reads was more uniform.

(I). The proportion of raw counts for the barcode of each sample in the sequencing library. Top panel: 32MIX for manual operation; Bottom panel: 32MIX for automatic operation.


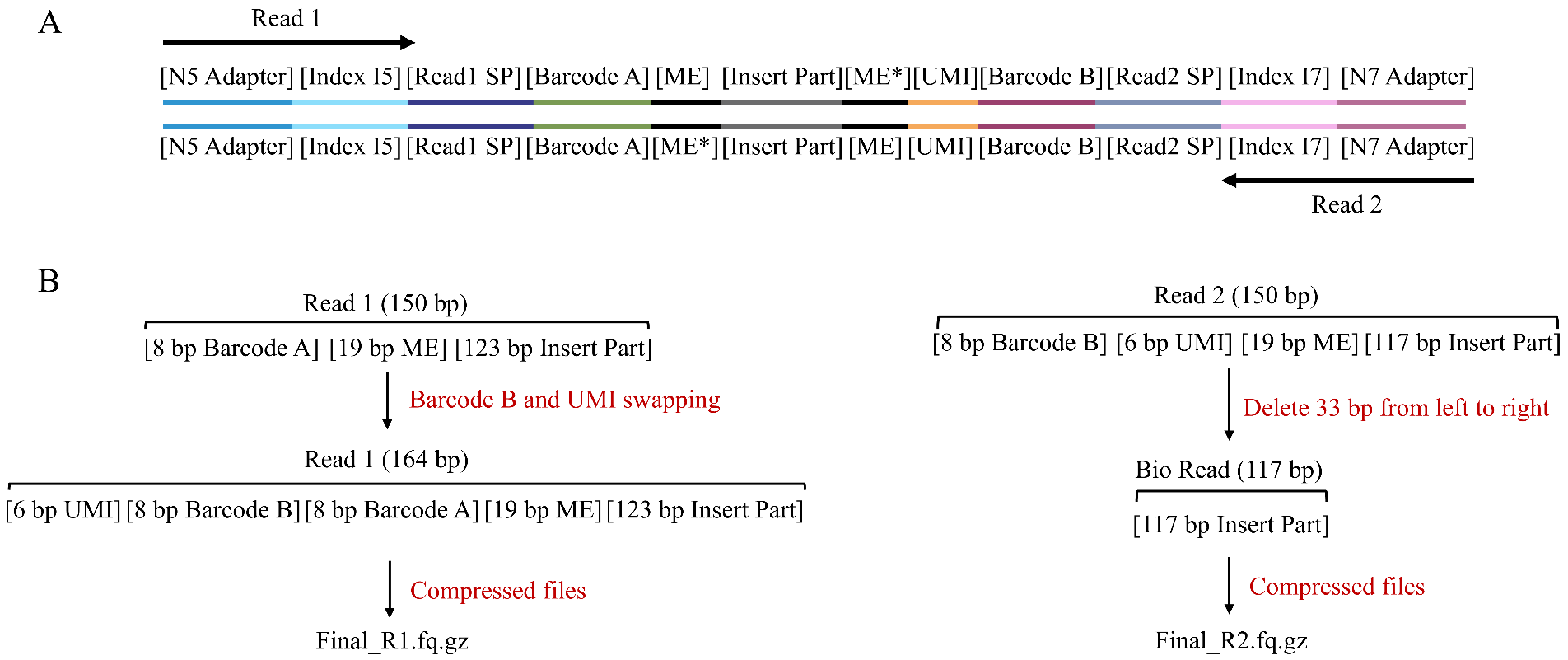


**Figure S15.** (A) Library structure diagram of CBTi-seq; (B) Schematic diagram of the barcode switching process of offline sequencing data.
